# Dynamic Analysis of Gene Expression and Genome-wide Transcription Factor Binding during Lineage Specification of Multipotent Progenitors

**DOI:** 10.1016/j.stem.2013.09.003

**Published:** 2013-12-05

**Authors:** Gillian May, Shamit Soneji, Alex J. Tipping, Jose Teles, Simon J. McGowan, Mengchu Wu, Yanping Guo, Cristina Fugazza, John Brown, Göran Karlsson, Cristina Pina, Victor Olariu, Stephen Taylor, Daniel G. Tenen, Carsten Peterson, Tariq Enver

**Affiliations:** 1 Stem Cell Group, UCL Cancer Institute, University College London, London WC1E 6BT, UK; 2 Molecular Haematology Unit, Weatherall Institute of Molecular Medicine, University of Oxford, Oxford OX3 9DS, UK; 3 Computational Biology and Biological Physics, Department of Theoretical Physics, Lund University, 223 62 Lund, Sweden; 4 Computational Biology Research Group, Weatherall Institute of Molecular Medicine, University of Oxford, Oxford OX3 9DS, UK; 5 Cancer Science Institute, National University of Singapore, Singapore 117599; 6 Harvard Stem Cell Institute, Harvard Medical School, Boston, MA 02115, USA

## Abstract

We used the paradigmatic GATA-PU.1 axis to explore, at the systems level, dynamic relationships between transcription factor (TF) binding and global gene expression programs as multipotent cells differentiate. We combined global ChIP-seq of GATA1, GATA2, and PU.1 with expression profiling during differentiation to erythroid and neutrophil lineages. Our analysis reveals (1) differential complexity of sequence motifs bound by GATA1, GATA2, and PU.1; (2) the scope and interplay of GATA1 and GATA2 programs within, and during transitions between, different cell compartments, and the extent of their hard-wiring by DNA motifs; (3) the potential to predict gene expression trajectories based on global associations between TF-binding data and target gene expression; and (4) how dynamic modeling of DNA-binding and gene expression data can be used to infer regulatory logic of TF circuitry. This rubric exemplifies the utility of this cross-platform resource for deconvoluting the complexity of transcriptional programs controlling stem/progenitor cell fate in hematopoiesis.

## Highlights

• Cross-platform resource for TF-network regulation of multipotent blood cell fate
• DNA motif dependence and changing specificity of GATA factors in lineage choice
• Modeling-based inference identifies GATA2 repression of PU.1 in multipotent cells
• Priming, recruitment, and switching modes of GATA interplay during differentiation

## Teaser

A systems-level resource of the GATA-PU.1 axis provides insight into the dynamics of transcriptional programs during hematopoietic lineage commitment and differentiation.

## Introduction

Transcription factors (TFs) are key regulators of stem and progenitor cell fates. Hematopoiesis provides a model to study TF-mediated regulation of cell fate (Orkin and Zon, 2008), with enforced expression of TFs in both multipotent and lineage-committed progenitors demonstrating their capacity to influence, instruct, or redirect cell fate. Such studies inform the programming and reprogramming of embryonic stem and somatic cells using lineage- or stem cell-affiliated TFs (Graf, 2011; Graf and Enver, 2009).

TFs presumably regulate fate by modulating transcriptional networks (Rothenberg and Anderson, 2002; Swiers et al., 2006). Although small regulatory modules have been derived by combining gene expression data with computational and functional analysis of *cis*-regulatory elements (Basso et al., 2005; Boyer et al., 2005; Donaldson et al., 2005; Loh et al., 2006; Novershtern et al., 2011; Suzuki et al., 2009), understanding global transcriptional regulation remains a challenge. ChIP-seq allows genome-wide mapping of TF binding and provides “hard-wiring” of transcriptional networks, but unambiguous linkage of genome-wide TF binding to global gene expression has not yet been achieved. This reflects the complexity observed, with individual genes being regulated by multiple TFs at multiple regulatory elements and differential regulation in different cell compartments.

The distinctive transcriptional profiles of different hematopoietic compartments (see Kee, 2011 for an overview) imply significant changes in TF binding as cells undergo lineage commitment and differentiation. Genome-wide targets have recently been described for many hematopoietic TFs (see Hannah et al., 2011), but studies have generally focused on binding within a single compartment, precluding appraisal of the lineage specificity of interactions and how cistromes change across commitment boundaries.

The hematopoietic TFs GATA1, GATA2, and PU.1 provide an attractive trio for dissecting differentiation, due to their importance as key regulators of hematopoiesis (reviewed in Doré and Crispino, 2011; Gupta et al., 2009; Suzuki et al., 2011) and their dynamic expression; GATA2 is associated primarily with stem cells and multipotent progenitors, GATA1 with erythroid cells and megakaryocytes, and PU.1 with myeloid and lymphoid cells. These TFs have been widely studied within differentiated cells, but how the lineage-affiliated programs that they drive evolve from the multipotent ground state remains unclear. Several recent studies have described their genome-wide targets (reviewed in Doré and Crispino, 2011). Valuable though these studies have been, they are largely nondynamic, and have not encompassed the stage- and lineage-specific complexity, and cross-regulatory interactions, of these TFs. The latter is exemplified by GATA switching, the replacement of GATA2 by GATA1 during erythroid differentiation, which has served as a paradigm for how changes in TF binding may both control and reflect lineage-specific commitment and differentiation (Bresnick et al., 2010; Kaneko et al., 2010).

Using the FDCPmix model system, we generated global gene expression profiles throughout the unilineage specification and differentiation of hematopoietic multipotent cells (MPCs) to erythroid and neutrophil cells, complemented by gene expression profiling of comparable primary cell compartments prospectively isolated from mouse bone marrow. We also performed ChIP-seq of GATA1, GATA2, and PU.1 in both multipotent and differentiated FDCPmix cells. We use this dynamic gene expression and TF-binding data to (1) provide a comprehensive description of multipotent progenitor, erythroid, and neutrophil cell gene expression and the genome-wide targets of GATA1, GATA2, and PU.1; (2) provide high-resolution global gene expression data for MPCs undergoing lineage specification; (3) dissect how the programs regulated by GATA1 and GATA2 relate to each other and are impacted by DNA sequence; (4) relate combinatorial binding patterns and/or DNA motifs to gene expression; and (5) infer the nature of regulatory interactions between GATA1, GATA2, and PU.1 through dynamic modeling. The data have been compiled into a queryable MySQL resource for the hematopoietic, stem cell, and bioinformatic research communities (see Experimental Procedures for access details and Supplemental Experimental Procedures available online for further information). A greater understanding of the function of key regulatory TFs within a well-characterized system such as hematopoiesis should provide broader insights into how transcriptional programs and networks interact to control lineage commitment and differentiation.

## Results

### Genome-wide Analysis of a Dynamic Differentiation System

**Figure 1:**
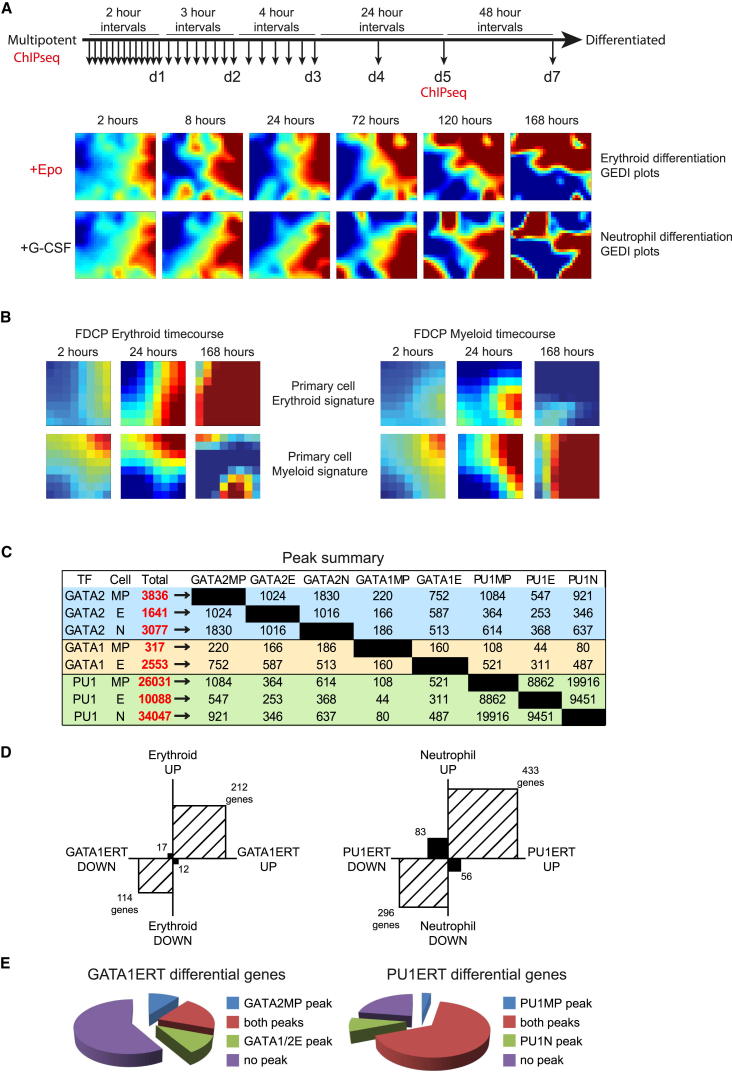
Dissecting Hematopoietic Differentiation (A) Erythroid and neutrophil differentiation time course of FDCPmix cells. Time points for RNA (arrows) and ChIP-seq analysis are indicated. GEDI (Eichler et al., 2003) plots show changes in global transcriptomes through erythroid (upper) and neutrophil (lower) differentiation. Each pixel represents a group of coexpressed genes. (B) Behavior of primary murine hematopoietic erythroid and myeloid signature genes in FDCPmix erythroid (left) and neutrophil (right) time courses. (C) Peaks identified in each ChIP-seq experiment with their pairwise overlaps. MP, multipotent cells; E, erythroid cells (day 5); N, neutrophils (day 5). (D) Genes modulated 2-fold by GATA1ERT induction in MP cells (left) were scored for up- or downregulation (d7/d0 >2 or <0.5) in the erythroid time course. The plot shows the number of genes with concordant (hatched) or discordant (black) regulation in the two experiments. PU.1ERT responses were compared against the neutrophil time course (right). See also Figure S2B. (E) Left: GATA1ERT-responsive genes (as in D) with four GATA factor-binding profiles: bound only by GATA2 in MP cells (GATA2MP); bound by GATA2 in MP cells and GATA1 or GATA2 in E cells (both peaks); bound by GATA1 or GATA2 in E but not MP cells (GATA1/2E); and not bound (no peak). Right: PU.1ERT-responsive genes bound by PU.1 in MP cells (PU1MP), neutrophils (PU1N), or both. See also Figures S1 and S2.

We used FDCPmix cells as a model system to study cell-fate choice, combining gene expression, global TF binding, and TF perturbation data to provide a multiplatform resource. FDCPmix cells are karyotypically normal and nonleukemogenic, self-renew in IL-3, and differentiate in response to physiological cues (Spooncer et al., 1986). We generated a high-density time course of gene expression during 7 days of erythroid (E) and neutrophil (N) differentiation, with sampling concentrated over the first 72 hr (Figure 1A). Differentiation was evaluated by morphological analysis (Figure S1A). Transcriptional divergence between the E and N lineages was discernible after 2 hr, with clear differences evident by 72 hr (Figure 1A). To compare FDCPmix cells with normal murine bone marrow cells, we analyzed global gene expression in multipotent progenitors (Kit^+^Lin^−^Sca1^+^; KLS) plus three stages of erythroid and myeloid cell differentiation, and derived primary erythroid and myeloid expression signatures. Global gene expression was broadly comparable in FDCPmix, primary murine cells (Figure 1B), and primary human cells (Novershtern et al., 2011) (Figures S1B and S1C), both validating the FDCPmix model and confirming conservation of transcription between mouse and human hematopoiesis.

We next performed global ChIP-seq of GATA1, GATA2, and PU.1 in multipotent progenitor cells and committed erythroid and neutrophil cells after day 5 of differentiation. Gene set enrichment analysis identified MP, and E and N (day 5) cells as corresponding most closely to the KLS, colony-forming unit erythroid, and GMP compartments of primary murine bone marrow, respectively (Figure S2A). Preliminary analysis shows how the binding profiles of these TFs overlap and evolve as cells differentiate (Figure 1C). Lineage-associated TF binding is often initiated in MPCs, consistent with lineage priming. Salient points include: (1) 58% of locations bound by PU.1 in neutrophils and 88% of locations bound in erythroid cells are also bound in MPCs, suggesting much of the PU.1-driven lineage programs are initiated in the MP compartment; 24% (4,787 peaks) of neutrophil PU.1-binding events that are “primed” in MPCs are lost during erythroid differentiation, attesting to their lineage specificity (not shown); (2) peaks bound by GATA2 in MPCs persist more often in N than in E cells (48% versus 27%), consistent with a perhaps underappreciated role for GATA2 in the neutrophil lineage; note that in erythroid-committed cells, unlike neutrophils, both GATA1 and GATA2 contribute to overall GATA factor activity; and (3) GATA2 and PU.1 binding overlap substantially in MPCs, with a total of 1,084 shared locations (28% of GATA2 MPC sites but only 4% of the larger PU.1 data set). The extensive binding of PU.1 in E cells is consistent with reports of an erythroid role for PU.1 (Wontakal et al., 2011); the observation that much of the binding originates in MPCs provides a developmental context.

To place the FDCPmix ChIP-seq data into context, we compared them with data published for GATA1, GATA2, and PU.1 in roughly comparable cell types. Despite different experimental and data analyses, between 33% and 57% of peaks detected in FDCPmix were also present in the most relevant of the published data sets (Figure S1D).

Finally, transcriptional programs elicited by cytokine-mediated differentiation were compared to those triggered by activation of inducible GATA1 and PU.1 moieties in MPCs. Gene expression changes induced by GATA1ERT and PU.1ERT broadly recapitulated those seen during E and N differentiation, respectively (Figure 1D; Figure S2B), with 61% of GATA1ERT-upregulated genes and 40% of PU.1ERT-upregulated genes also being upregulated 2-fold after 7 days of differentiation. GATA1ERT- and PU.1ERT-induced changes were also largely consistent with gene expression changes seen in early erythroid and myeloid differentiation of primary cells (Figure S2C). Around one-third of GATA1ERT-responsive genes were associated with binding of GATA1 in E cells (Figure 1E), as reported in similar studies (Fujiwara et al., 2009; Welch et al., 2004; Yu et al., 2009), whereas around three-quarters of PU.1ERT-responsive genes were bound by PU.1 in MP and/or N cells.

We used this resource to dissect the interplay of GATA1, GATA2, and PU.1, exemplifying ways of using these data to provide insights into TF-mediated regulation of cell identity. In particular, we exploited the combinatorial nature of the data to stratify each global ChIP-seq data set into more coherent subsets, enabling linkage of TF binding to gene expression and DNA motif content.

### GATA1 and GATA2 Have Different DNA Sequence Preferences

**Figure 2:**
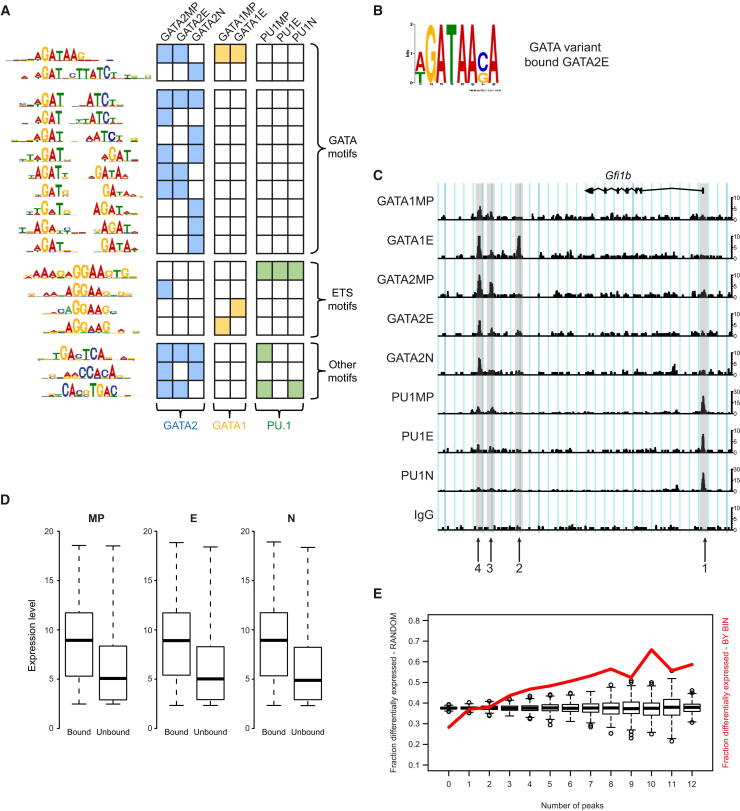
Motif Discovery and Global Binding Behaviors (A) De novo detection of DNA sequence motifs by CisFinder. ChIP-seq experiments are grouped by TF; blue, yellow, and green boxes denote motifs enriched by GATA2, GATA1, and PU.1, respectively. (B) GATA motif variant identified by MEME as bound by GATA2 in E cells. (C) Binding over the *Gfi1b* locus in eight ChIP-seq experiments versus IgG control. Arrows indicate four locations with different TF-binding profiles. (D) In multipotent, erythroid, and neutrophil cells, median expression levels of genes bound by any of the three TFs analyzed are higher than for unbound genes. All differences between median expression values (bound versus unbound) are significant (p > 2.6 × 10^−16^). Whiskers depict the most extreme data points. (E) Genes were binned according to the total number of bound regions associated with them in the eight ChIP-seq experiments, and the fraction of differentially expressed genes in each bin is plotted (red line). Box plots show the fraction of differentially expressed genes within randomly selected bins of the same size. Whisker length is defined as 1.5× interquartile range. See also Figure S3.

We first explored the in vivo DNA sequence preferences of GATA1 and GATA2. The prevailing view is that the DNA-binding properties of these TFs, which have highly related zinc fingers, are essentially identical (Bresnick et al., 2010). CisFinder and MEME identified AGATAAG as a consensus motif for both factors (Figure 2A; Figure S3A), refining the prevailing consensus GATA motifs of SWGATAAVV (Fujiwara et al., 2009) and WGATAR (Tijssen et al., 2011; Wilson et al., 2010). Strikingly, GATA2—but not GATA1—also enriched novel GATA-related motifs, including several repeat forms of WGAT in direct and palindromic configurations spaced by 3–4 and 3–5 bp, respectively. Motif usage by GATA2 varied with cell type: in neutrophils, the GATA repeats/palindromes scored highly, whereas in MP and E cells both WGATAAG and subsets of the repeats/palindromes were enriched. MEME also detected GATA2 binding to a further GATA variant, wGATAAsA, in E cells (Figure 2B). For PU.1, an extended ETS consensus motif of GGAAGTG was identified; inclusion of less conserved flanking nucleotides extends this to (AAAGA)GGAAGTG (Figure 2A; Figure S3A), matching the PU.1 consensus derived in B cells and macrophages (Heinz et al., 2010).

Other enriched motifs included simple ETS (GGAAG), AP-1/NF-E2/MAF (TGASTCA), RUNX1 (CCACA), and MYC (CACGTGAC) consensus motifs, consistent with previous reports of GATA-ETS (Pimanda et al., 2007) and AP1-GATA2 interactions, and enrichment of RUNX motifs by GATA1 and SCL in megakaryocytes (Tijssen et al., 2011). E box-GATA composite motifs—important in erythroid cells (Kassouf et al., 2010; Vyas et al., 1999)—were not identified, although a canonical SCL-like E box (CWGCWGC) was enriched by GATA1 in MPCs (Figure S3A).

Overall, these data demonstrate (1) differences between GATA1 and GATA2 DNA sequence preferences in vivo and (2) how the spectrum of sequences bound by GATA2 shifts as cells undergo differentiation, contrasting with the uniformity of PU.1 binding.

### TF-Binding Complexity Predicts Differential Gene Expression

Complexity of TF binding is exemplified by the *Gfi1b* locus (Figure 2C), demonstrating (1) multiple TF-bound regions, (2) simultaneous binding of a TF to more than one region, and (3) dynamic changes in TF binding on differentiation. *Gfi1b* is not atypical, either in terms of the number of peaks or the degree of TF interchange on differentiation, as judged by a dynamic binding complexity scoring matrix (see Figure S3B; Supplemental Experimental Procedures). Genes bound by GATA1, GATA2, or PU.1 were generally expressed at higher levels than genes not bound (Figure 2D), implying that these TFs contribute positively to regulation of a large proportion of MP, E, and N cell transcriptomes. Genes associated with more regulatory elements were more likely to be differentially expressed (Figure 2E), presumably because this allows for more combinatorial TF binding and regulation.

### Dynamic Interplay of GATA1 and GATA2 Transcriptional Programs

**Figure 3:**
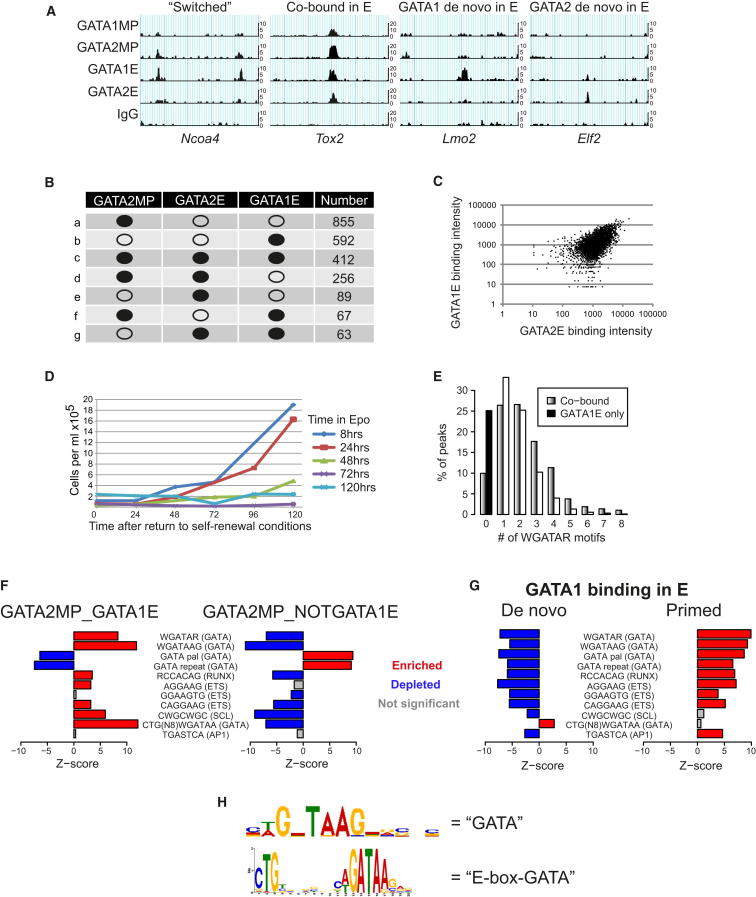
Dynamism of GATA Factor Binding during Erythroid Differentiation (A) Examples of different modes of GATA binding in MP and E cells. GATA2 binding in MP cells can be lost when GATA1 binds (Switched) or is retained (Co-bound in E). GATA1 and GATA2 also bind in (E) without prior binding of GATA2 in MP cells (de novo in E). (B) Frequency of different GATA factor-binding profiles (a–g) in MP and E cells, ordered by decreasing frequency. Filled circles, stringently bound; open circles, stringently not bound. (C) Quantitation of GATA2 (x axis) and GATA1 (y axis) binding in erythroid cells. Data points show the tag number (log scale) scored in GATA2E and GATA1E ChIP-seq experiments at sites originally bound by GATA2 in MP cells. (D) Cytokine-switching experiments. FDCPmix cells were incubated in erythroid differentiation conditions for 8, 24, 48, 72, or 120 hr, and then washed and returned to self-renewal conditions (IL-3). Viable cell counts were performed each day for 5 days using trypan blue exclusion. Cells preexposed to erythroid differentiation conditions for 2 days or more failed to expand in response to IL-3. (E) Frequency of WGATAR motifs within peaks cobound by GATA1 and GATA2 (gray bars) or bound by GATA1 alone (black bars) in erythroid cells. (F) Motifs enriched/depleted in GATA2MP peaks that do (left) or do not (right) bind GATA1 in erythroid cells. Motifs enriched or depleted (z scores) with FDR <0.05 are shaded red and blue, respectively; insignificant enrichments/depletions are shaded gray. Consensus motif designations are shown in parentheses. (G) Motifs enriched/depleted within the subset of GATA1E peaks that are de novo bound by GATA1 (left) or GATA2 primed in MP cells (right). Colors are as in (F). (H) Motif analysis of de novo GATA1E peaks in isolation identifies a novel degenerate GATA and an E box-GATA motif not detected by analysis of all GATA1E peaks. See also Figure S4.

The interchange or “switching” of GATA factors in experimental systems of erythroid maturation has provided a plausible paradigm for erythroid specification of MPCs. We tested this through direct comparison of the GATA1 and GATA2 cistromes in MP and E cells. Figure 3A shows peaks with four distinct patterns of GATA1 and GATA2 binding, demonstrating the complexity of GATA factor interplay. Using stringent criteria to define “bound” and “not-bound” locations (see Experimental Procedures), the most common pattern observed was the binding of GATA2 in MPCs but neither factor in E cells (Figure 3B, profile a), followed by just binding of GATA1 in E cells (profile b). Surprisingly few GATA2 MPC peaks underwent GATA switching (profile f); this occurs at less than 2% of all GATA2 MPC peaks, and visual inspection reveals that even these tend to display somewhat incomplete switching. In fact, many of the locations bound by GATA2 in MPCs were bound by both GATA2 and GATA1 in E cells (profile c). A displacement model of GATA switching predicts that a strong signal in erythroid cells for one GATA factor would be accompanied by a relatively weak signal for the other. Contrary to this, enumeration of the sequence tags as a measure of occupancy revealed an overall trend where stronger binding of GATA2 in erythroid cells was associated with stronger binding of GATA1, and vice versa (Figure 3C).

Cytokine-switching experiments demonstrated that the vast majority of cells underwent irreversible erythroid commitment between 24 and 48 hr of differentiation, as judged by the inability of the cells to resume self-renewal in response to IL-3 (Figure 3D). Combined with the erythroid morphology of the cells (Figure S1A), we conclude that the bulk of the erythroid (day 5) cells used for ChIP-seq analysis has undergone erythroid lineage commitment and that this can, therefore, occur in the absence of widespread GATA switching.

Peaks shared by GATA1 and GATA2 in erythroid cells had a higher frequency of multiple WGATAR motifs than those bound by just GATA1 (Figure 3E); 76% contained more than one WGATAR and/or GATA repeat/palindrome, and all GATA motifs were enriched (Figure S4A). This suggests a mode of GATA factor interplay whereby GATA2 binding in MPCs persists in E cells and acts as a “pioneer” for binding of GATA1 to a second GATA motif. This provides a developmental context for the sharing of sites by GATA1 and GATA2 reported in human erythroid cells (Fujiwara et al., 2009), and indicates that GATA2 and GATA1 cooperate extensively to regulate erythroid differentiation.

GATA2 also binds de novo in E cells to a number of locations (Figure 3B, profile e), and some GATA2 erythroid peaks that persist from MPCs fail to bind GATA1 (Figure 3B, profile d). This indicates an erythroid role for GATA2 distinct from its role in MPCs. Most de novo GATA2 peaks display weak binding of GATA1 (not shown); thus, de novo GATA2E binding may reflect an intermediate stage of the erythroid program, where sites that are not primed by GATA2 in MPCs sequentially bind GATA2 and then GATA1 as their expression increases during differentiation (Figure S4B).

Analysis of DNA motifs suggests one mechanism for selective recruitment of GATA factors to particular sites. Where GATA2 is bound in MPCs, recruitment of GATA1 is favored by the presence of WGATAAG/WGATAR (Figure 3F). Specifically, peaks containing the WGATAAG sequence(s) and lacking GATA repeats/palindromes were four times more likely to recruit GATA1 than peaks that contain GATA repeats/palindromes but lack WGATAAG/WGATAR (39% versus 10%) (not shown). RUNX, E box, and ETS motifs also favor the binding of GATA1, pointing to accessory TFs likely to influence the GATA-regulated program.

Motifs also influence de novo erythroid binding of GATA1 and GATA2. De novo GATA1 peaks (Figure 3A; Figure S4C) were unexpectedly depleted for all GATA motifs tested (Figure 3G), although motif discovery on these peaks in isolation identified a degenerate GATA motif WGNTAAG and a composite half-E box-GATA motif (CTGN_8_WGATAA) (Figure 3H). The latter was also reported in SCL-GATA-cobound sequences in erythroid cells (Kassouf et al., 2010; Soler et al., 2010). Enrichment of this motif specifically within de novo GATA1 E peaks is consistent with SCL functioning independently of DNA binding when participating in early aspects of the GATA program (Kassouf et al., 2008; Porcher et al., 1999). In contrast, de novo GATA2 erythroid peaks showed no specific enrichment or depletion of any GATA motif (not shown).

**Figure 4:**
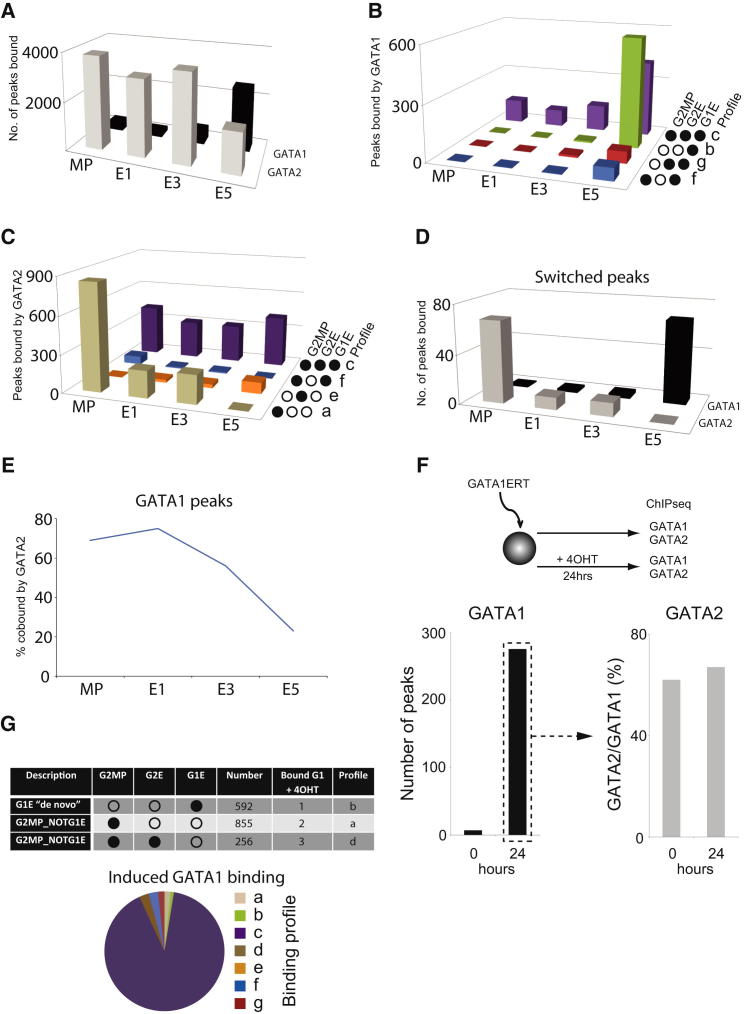
GATA2 as a Pioneer Factor for GATA1 (A) Number of GATA2 and GATA1 peaks in MP cells and after 1, 3, and 5 days of erythroid differentiation (E1, E3, E5). (B) Binding of GATA1 at intermediate time points to regions previously classified by their GATA binding in MPC and E5 cells (see Figure 3B). MP, E1, E3, and E5 as in (A). (C) GATA2 binding at intermediate time points to regions previously classified by their GATA binding in MPC and E5 cells (see Figure 3B). MP, E1, E3, and E5 as in (A). (D) Loss of GATA2 and gain of GATA1 through erythroid differentiation at peaks defined as “switched.” MP, E1, E3, and E5 as in (A). (E) Percentage of GATA1-bound regions cobound by GATA2 at four stages of erythroid differentiation. (F) ChIP-seq analysis of GATA1 binding in multipotent cells, using tamoxifen activation of GATA1ERT (4OHT, 24 hr). Regions bound by GATA1 after induction (left; 24 hr) were reanalyzed for GATA2 binding before and after induction (right; 0 and 24 hr). (G) GATA1 binding in induced cells versus normal erythroid differentiation. Upper: regions defined as profiles b, a, and d according to their binding in MPC and E5 cells fail to induce binding in GATA1 in multipotent cells. Lower: regions bound by GATA1 after induction that correspond to profiles a–g (Figure 3B) fall mainly into profile c (bound by GATA2 in MPC and by both GATA2 and GATA1 in E5 cells).

The evolution of the GATA program and the role of GATA2 in recruiting GATA1 were further explored by generating additional ChIP-seq data from days 1 and 3 of erythroid differentiation (E1 and E3) and combining them with the MPC and E data (here termed E5). Most changes in GATA binding occurred between days 3 and 5 of differentiation (Figure 4A). However, the simple trend of reduced GATA2 and increased GATA1 binding disguises multiple different behaviors. Regions classified by their GATA-binding profiles in MPC and E5 (see Figure 3B) display different timing of GATA1 acquisition (Figure 4B). The majority of regions bind GATA1 only after day 3 (profiles f, g, and b); the exception is peaks that are primed by and retain GATA2 (profile c), some of which are also cobound by GATA1 in MP and/or early erythroid cells. GATA2-binding dynamics also varies (Figure 4C); most GATA2MP peaks that lose GATA2 do so during day 1 of differentiation (profiles a and f), at the same time as “de novo” GATA2 peaks are emerging (profile e) and other regions are retaining GATA2 (profile c). The contrasting behaviors of GATA2 and GATA1 at regions previously defined as “switched” (profile f) are shown in Figure 4D.

In MP, E1, and E3 cells, most GATA1 binding is at sites also bound by GATA2 (Figure 4E), consistent with the notion that GATA2 facilitates binding of GATA1. We directly tested this through ChIP-seq of GATA1ERT, to determine where GATA1 can bind when forcibly expressed in an essentially multipotent cell environment and how this relates to GATA2 occupancy. GATA1 binding increased sharply after induction (Figure 4F, left), mainly at sites bound by GATA2 both prior to and after induction (Figure 4F, right, 0 hr, and 24 hr). In E5 cells, GATA1 is bound to both primed and de novo sites, obscuring whether this GATA2 cobinding facilitates GATA1 binding or simply accompanies it. Crucially, when forcibly expressed in a multipotent cell, GATA1 failed to bind all but one of the 592 regions previously defined as de novo GATA1 bound in E5 cells (Figure 4G, profile b). Thus, GATA1 is unable to bind these sites even while simultaneously binding strongly at GATA2-bound regions; the simplest explanation is that pioneering by GATA2 is a critical determinant of GATA1 recruitment. However, GATA1 does not bind indiscriminately wherever GATA2 is bound; regions that do not normally recruit GATA1 in E5 cells (Figure 4G, profiles a and d) also failed to bind induced GATA1. Thus, other local features of the multipotent cell environment must hinder GATA1 recruitment at these sites. Induced GATA1 binds mostly to regions primed by GATA2 in MPCs and bound by both GATA2 and GATA1 in E5 cells (Figure 4G, pie chart, profile c). Overall, it seems that, when GATA1 is expressed normally in early erythroid cells or forcibly expressed in multipotent cells, its binding is restricted to regions that are bound by GATA2, strongly supporting the proposed role of GATA2 as a pioneer for GATA1.

### Linking Gene Expression to TF Binding and DNA Motifs

**Figure 5:**
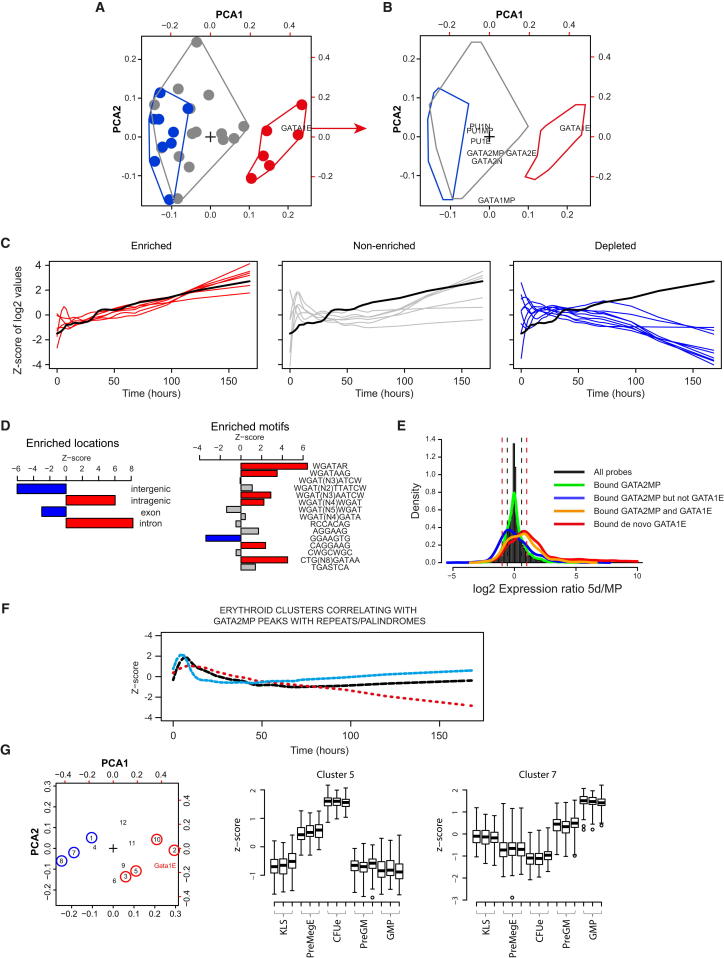
Linking TF Binding and DNA Motifs to Gene Expression (A) Correspondence analysis of bound genes versus erythroid gene expression clusters (see Figures S5A and S5B) showing location of expression clusters (filled circles) and GATA1E-bound genes (GATA1E) relative to the point of inertia (cross). The distal location of the GATA1E-bound genes indicates significant association with particular gene expression clusters. Enriched and depleted clusters (FDR <0.05) are colored red and blue, respectively; associations with gray expression clusters were nonsignificant. PCA, principal-component analysis. (B) Correspondence analysis of all ChIP-seq data sets. The zones occupied by the gene expression clusters in (A) are indicated, with the colors continuing to represent their enrichment/depletion in GATA1E-bound genes. Data sets lying closer to the point of inertia have less significant associations with expression clusters. (C) Erythroid expression of GATA1 (black line) versus erythroid expression cluster centroids. Left: clusters enriched for GATA1E binding (clusters 7, 14, 16, 25, 26). Middle: upregulated clusters not enriched for GATA1E binding (clusters 3, 11, 20, 21, 22, 30). Right: clusters depleted for GATA1E binding (1, 4, 10, 13, 17, 18, 27, 28, 29). Clusters are colored as in (A). (D) GATA1E-bound regions in clusters significantly associated with GATA1E binding are enriched for an intronic location (left) and have a biased motif content (right) compared to all GATA1E-bound genes. (E) Erythroid expression of genes with different GATA-binding profiles. The histogram shows the fold change for all probes between day 0 (MP) and day 5 of erythroid differentiation (All probes); density indicates the number of genes; dotted lines indicate 1.5- and 2-fold up/downregulation; line graphs show the fold change for genes bound/unbound in the indicated experiments. (F) GATA2MP peaks split by DNA motif are associated with different erythroid expression patterns. Centroids of three clusters (3, black; 10, red; 19, blue) enriched in GATA2MP peaks with WGAT repeats and palindromes. (G) Left: correspondence analysis of FDCPmix TF binding versus primary hematopoietic gene expression clusters, showing the location of GATA1E, which again showed the most significant associations and lay farthest from the point of inertia. Numbers represent expression clusters; clusters enriched or depleted for GATA1E binding are circled red or blue, respectively. Expression (z score) in primary hematopoietic cells is shown for the gene clusters most enriched (middle) and most depleted (right) for GATA1E binding. Whisker length is defined as 3× interquartile range. See also Figure S5 and Tables S1, S2, S3, S4, and S5.

We next used an unsupervised approach to identify significant associations between genome-wide TF-binding data and gene expression. We used correspondence analysis to rapidly visualize the associations between ChIP-seq data and gene expression (Figure 5A). Simultaneous global analysis of the eight original ChIP-seq data sets against 60 clusters of genes coexpressed during E or N differentiation (30 for each lineage; see Figures S5A and S5B) reveals that only GATA1 binding in erythroid cells (GATA1E) is peripherally located relative to the point of inertia (black cross), indicating significant associations of this data set with particular gene expression clusters (filled circles); clusters enriched or depleted for GATA1 binding (see Table S1) are colored red and blue, respectively. In contrast, GATA2 and PU.1 ChIP-seq data sets fall near the point of inertia (Figure 5B), indicating generally weak associations with the expression clusters.

Strikingly, the expression profiles of all clusters enriched for GATA1E binding followed that of GATA1 itself (Figure 5C, left). Clusters upregulated but showing poorer correlation with GATA1 expression were not enriched for GATA1 binding (Figure 5C, middle), and clusters depleted for GATA1 binding were downregulated (Figure 5C, right). Binding of GATA1 within the enriched *Gata1*-correlated genes was strongly biased toward an intronic location with further enrichment of WGATAR/WGATAAG and E box-GATA motifs and depletion of the PU.1 ETS motif (GGAAGTG) (Figure 5D). This approach was less informative for PU.1; nevertheless, PU.1 binding in neutrophils was strongly associated with three upregulated neutrophil expression clusters (Figure S5C). These clusters were also associated with PU.1 in both MP and E cells (Table S1), consistent with our initial observation that many sites are bound by PU.1 in all three cell types. Interestingly, two of these clusters were also associated with GATA2 binding in neutrophils (Table S1), hinting at coregulation by GATA2 and PU.1 in this lineage.

Initial analysis of GATA2 binding in MPCs failed to show any significant associations with erythroid expression clusters (Figure S5D, left). This was confirmed by the observation that genes bound by GATA2 in MPCs display diverse expression behaviors during erythroid differentiation, matching the distribution seen for all genes (Figure 5E, green). Stratifying the peaks according to whether they subsequently bind GATA1 does, however, help predict expression trajectories. GATA2MP-bound elements that recruit GATA1 in E cells are biased toward upregulation (Figure 5E, orange), whereas those that fail to recruit GATA1 are biased toward downregulation (Figure 5E, blue).

We further dissected GATA2MP binding according to TF interplay and DNA motif content. GATA2MP peaks that recruit GATA1 were associated with two erythroid-upregulated clusters that had already been associated with GATA1E binding (Table S2, clusters 7 and 16). Subdividing GATA2MP peaks by motif showed that GATA repeats/palindromes were associated with clusters that were broadly flat or downregulated during erythroid differentiation (Figure 5F; Table S3), in contrast to the association of GATA1 with upregulated clusters. GATA2MP peaks split by motif were also associated with various neutrophil expression clusters (Table S4).

Together, these analyses exemplify how this FDCPmix resource can be used to identify associations between genome-wide TF-binding data and gene expression trajectories. In principle, this could be repeated in primary hematopoietic progenitors, but ChIP-seq in these cells remains problematic due to their scarcity. However, the results obtained here have currency in primary cells because global cross-comparison of FDCPmix ChIP-seq data and primary cell gene expression clusters yielded similar conclusions (Figure 5G; Table S5). Thus, binding of GATA1 in FDCPmix E cells is positively associated with gene expression clusters that are upregulated in primary erythroid cells and negatively associated with downregulated clusters.

### Modeling Regulatory Interactions from Dynamic ChIP-Seq and Gene Expression Data

Establishing the regulatory architecture and behavior of TF circuits remains a significant challenge in systems biology. We used our data resource for dynamic modeling to infer the regulatory interactions between GATA1, GATA2, and PU.1, and tested its predictions within the same cell system.

**Figure 6:**
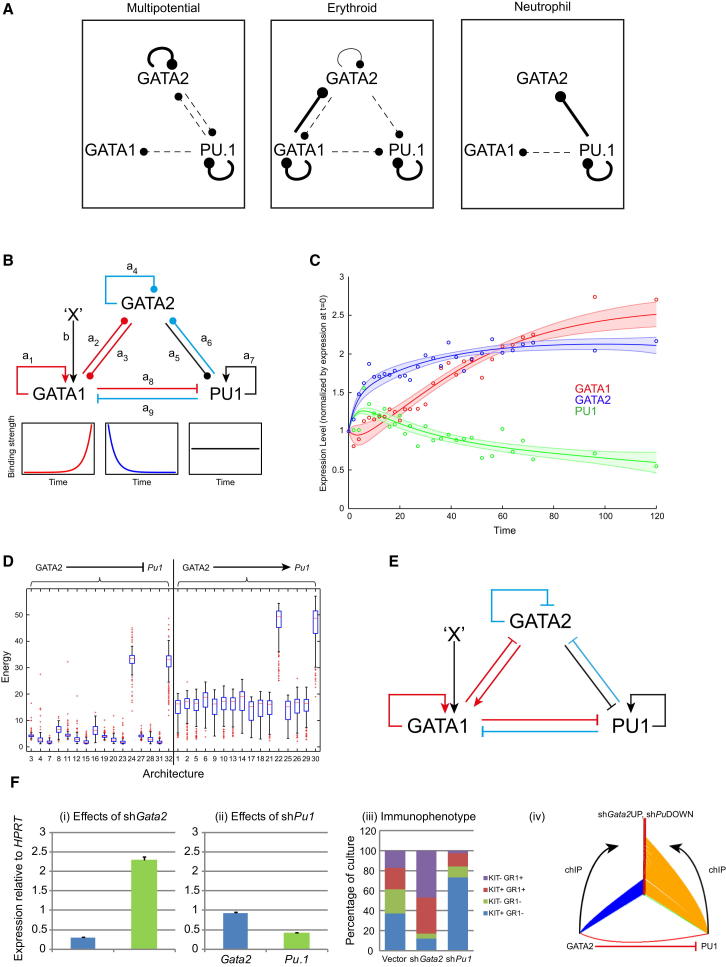
Modeling the GATA1, GATA2, and PU.1 Triad (A) Binding summary for GATA1, GATA2, and PU.1 over their own and each other’s loci, based on the ChIP-seq data. Bold, solid, and dotted connectors indicate strong, intermediate, and weak enrichments, respectively. See also Figures S6A and S6B. (B) Basal architecture for the triad during erythroid differentiation based on binding data in (A) and the literature. Binding strengths (a_x_, b) were modeled as exponentially increasing (red), exponentially decreasing (blue), or constant (black), according to the changes observed between MP and E cells. Circled arrowheads, interactions of unknown sign based solely on DNA-binding data; bent arrows and blunt arrowheads, positive autoregulation and cross-inhibition of GATA1 and PU.1 as reported in the literature. X represents an undefined, but predicted, constant positive input to *Gata1*. (C) Example of erythroid time course gene expression profile fits using the 60 best solutions for architecture 4 (see Table S6; Supplemental Experimental Procedures). Full lines, mean simulated expression; shaded contours, standard deviation; circles, experimental data points; red, *Gata1*; blue, *Gata2*; green, *Pu.1*. (D) Energies for all 32 possible networks (Table S6), corresponding to the 200 parameter sets and grouped according to the sign of the GATA2-PU.1 interaction. Left: GATA2 represses *Pu.1*; right: GATA2 activates *Pu.1*. Whisker length is defined as 1.5× interquartile range. (E) Example of a low-energy network (architecture 4) that provides a good fit (see C) to the observed expression data. (F) Knockdown of *Gata2* and *Pu*.1 in multipotent FDCPmix. Real-time quantitative RT-PCR analysis of *Gata2* and *Pu.1* expression following shRNA knockdown of (i) *Gata2* or (ii) *Pu.1*. Expression normalized to *Hprt* and relative to the control vector, represented as mean ± SEM. (iii) Differentiation of MP cells assessed by surface antigen expression. sh*Gata2* increased generation of Gr-1^+^ myeloid cells (either kit^+^ or kit^−^), whereas sh*Pu.1* decreased myeloid output. (iv) Hive plot showing connectivity of GATA2 and PU.1 ChIP-seq to genes perturbed by sh*Gata2*/sh*Pu.1* in multipotent FDCPmix. Red points (y axis) denote genes upregulated during neutrophil differentiation that are upregulated by sh*Gata2* and downregulated by sh*Pu.1*. Blue and orange lines represent genes bound in MP cells by GATA2 and PU.1, respectively. The single red line represents upregulation of *Pu.1* by sh*Gata2*. See also Figure S6 and Table S6.

We first examined the binding of GATA1, GATA2, and PU.1 to their own and each other’s loci during differentiation, estimating relative binding strength from the peak heights in our ChIP-seq data (Figure 6A; Figure S6A). Notably, the strongest interactions are autoregulatory. Binding of GATA2 to its own locus is strongest in MPCs and diminishes in E cells, whereas GATA1 binds the *Gata1* locus in E cells but not in MPCs. PU.1 strongly binds its own locus in MP, E, and N cells.

We used this information to infer the regulatory interactions between these TFs through erythroid differentiation (described in more detail in Supplemental Experimental Procedures). The aim was to infer a circuit for the auto- and cross-regulatory interactions between GATA1, GATA2, and PU.1 that could simulate their expression profiles during erythroid differentiation of FDCPmix cells. A base architecture was constructed from their binding in MP and E cells, with binding strengths modeled as exponentially increasing, decreasing, or remaining constant over time (Figure 6B). This was supplemented with previously reported antagonistic and autoregulatory interactions of GATA1 and PU.1 (Chickarmane et al., 2009). To determine the regulatory logic of binding interactions involving GATA2, architectures were constructed representing all 32 possible combinations of positive and negative interactions of GATA2 with itself and with GATA1 and PU.1 (Figure 6B, interactions a_2_ to a_6_; see also Table S5). Parameters were optimized to minimize the differences (energies) between simulated and observed expression data for all three TFs. Some architectures reproduced the observed gene expression data remarkably well (e.g., architecture 4, Figure 6C), whereas for other architectures it was impossible to find parameter sets that generated a good fit (Figures S6B and S6C). GATA2 repression of *Pu.1* was a consistent feature of all good-fit (low-energy) configurations (Figure 6D), suggesting *Pu.1* repression by GATA2 is central to early erythroid differentiation. To our knowledge, this interaction has not previously been reported in MPCs, although it has been observed in GATA1 null erythromegakaryocytic cells (Chou et al., 2009; Huang et al., 2009) and in embryonic stem cells engineered to express GATA2 (Kitajima et al., 2006). A revised circuit including this interaction (derived from architecture 4) is shown (Figure 6E).

We tested the repression of *Pu.1* by GATA2 by knocking down *Gata2* in MPCs using a gene-specific shRNA. As predicted by the modeling, a reduction in *Gata2* led to an increase in *Pu.1* expression (Figure 6Fi), but *Pu.1* knockdown (KD) had no effect on *Gata2* expression (Figure 6Fii). *Gata2* KD also resulted in myeloid differentiation, with an increase in surface expression of the myeloid marker Gr-1, whereas *Pu.1* KD led to a decrease in Gr-1^+^ cells (Figure 6Fiii). This confirmed the prediction from the modeling: that expression of *Pu.1* in MPCs is negatively regulated by GATA2, with ChIP-seq indicating this may be a direct effect via binding of GATA2 to the *Pu.1* promoter (Figure S6D).

Finally, we looked for molecular evidence of global activation of a myeloid program following *Gata2* KD, and assessed to what extent this could be directly attributable to a loss of GATA2 binding or could be driven by a secondary increase in PU.1 binding. Genes upregulated following *Gata2* KD include *Csf1r*, *Csf2ra*, *Csf3r*, *Mpo*, *Cd52*, *Lyz1*, and *Lyz2* (Figure S6E). Figure 6Fiv shows a hive plot integrating global gene expression changes following *Gata2* or *Pu.1* KD with ChIP-seq and neutrophil gene expression data. Many of the neutrophilic genes upregulated in response to *Gata2* KD appear to be direct targets of PU.1 rather than of GATA2, suggesting that GATA2 repression of *Pu.1* in MPCs restrains initiation of a PU.1-driven program of myeloid differentiation.

## Discussion

This cross-platform resource provides many opportunities for integrating TF-binding and gene expression data to explore molecular mechanisms underlying changes in cell fate. The most extensive data set describes cytokine-directed differentiation of a multipotent cell model, but is complemented by analysis of primary murine hematopoietic compartments and transcription factor-driven differentiation. Good concordance of FDCPmix with primary cells confirms its utility as a hematopoietic cell model amenable to systems-level analysis, and encourages confidence that the networks discussed herein have relevance to primary hematopoietic cells. The stringently identified TF-binding interactions reported appear robust, and provide a starting point to extend focused TF studies into primary hematopoietic progenitors, where ChIP-seq remains technically challenging due to cell-number constraints. Although our resource affords gene discovery, particularly for early lineage regulators, and has revealed some TF-specific insights, we have primarily used systems-level approaches to illuminate more generalized aspects of TF-mediated gene regulation.

An overview of the data indicates that PU.1, GATA1, and GATA2 achieve differential target gene expression through different mechanisms. PU.1 expression and binding are relatively nondynamic, suggesting differential activity is achieved largely via recruitment of cofactors, as described in B cells and macrophages (Heinz et al., 2010). In contrast, the tissue specificity of GATA1 action derives primarily from erythroid restriction of its expression. GATA2, like PU.1, is expressed in MP, E, and N cells, but displays considerable differential DNA binding between compartments and gains further target gene discrimination through its interplay with GATA1. GATA2 bound to a wider spectrum of sequences in vivo than anticipated, binding to a range of GATA repeats and palindromes previously hinted at by some in vitro studies (Badis et al., 2009; Trainor et al., 2000), and challenging the current view of uniformity of GATA factor DNA sequence recognition. Combined with gene expression analyses, this provides evidence that differential GATA motif usage is a component of GATA-driven global transcriptional programs.

The interplay of GATA1 and GATA2 during erythroid differentiation is more intricate and dynamic than expected. Although other studies have described the binding of GATA1 in erythroid cells (Cheng et al., 2009; Fujiwara et al., 2009; Kassouf et al., 2010; Yu et al., 2009) and GATA2 in multipotent cells (Li et al., 2011; Wilson et al., 2010), to our knowledge, no other study has described and compared their genome-wide shifts in binding as cells undergo erythroid commitment and differentiation. Our data indicate that the bulk of the GATA1 and GATA2 programs are in fact independent of each other. Importantly, however, GATA2 also functions as a global pioneer for GATA1 during erythropoiesis, facilitating its binding to a subset of GATA-regulatory elements, influenced by the underlying DNA sequence. The extent of canonical GATA switching observed here was unexpectedly low. Evidence for GATA switching comes mainly from studies of the *Gata2* locus in an erythroid model system where GATA1 null erythroblasts are induced to differentiate by activation of an ectopic GATA1ERT fusion protein (Bresnick et al., 2010; Fujiwara et al., 2009) but provides a plausible mechanism for global regulation of erythroid gene expression; more extensive GATA switching has indeed recently been reported in GATA1 null megakaryocytes (Doré et al., 2012). However, GATA2 is highly expressed in the absence of GATA1 (Fujiwara et al., 1996; Weiss et al., 1994), and is repressed rapidly on activation of the GATA1ERT fusion protein; the importance of relative GATA levels in achieving stage-specific gene regulation has recently been discussed (Suzuki et al., 2011). The extensive sharing of sites reported here and in K562 cells (Fujiwara et al., 2009) suggests that retention of GATA2 during recruitment of GATA1 may be more typical than switching. Additional switching may occur later in erythroid maturation, but it is clear that erythroid commitment and substantial differentiation can occur in the absence of widespread GATA switching, consistent with the observation that GATA1 null cells can differentiate as far as proerythroblasts (Pevny et al., 1995).

Some ChIP-seq studies have focused on regulatory elements cobound by multiple TFs to simplify analysis of these large data sets (Tijssen et al., 2011; Wontakal et al., 2012). Our data allow multiple strategies for combinatorial analysis via (1) dynamism of binding of a single TF during differentiation, (2) combinatorial binding of multiple TFs within one compartment, (3) TF interchange between compartments, (4) DNA motif content, and (5) expression behavior of linked genes. Deconstruction of the genome-wide data into subsets with more coherent characteristics lends itself to an iterative approach, as features identified in a subset of bound regions or genes can be used for further stratification. As dynamic TF-binding data accumulate, the power of this type of combinatorial approach will increase, as demonstrated in *Drosophila*, where binding patterns of several TFs over successive developmental stages are predictive of spatiotemporal expression (Zinzen et al., 2009).

Stratification of binding data also helped identify enriched DNA motifs and link them to both TF-binding and gene expression information. This was most evident for the binding of GATA2 in MP cells. Considered in toto, GATA2 binding was not associated with any particular expression trajectory during erythroid differentiation, but stratification by DNA motifs linked binding of GATA2 at GATA repeat/palindrome sequences to downregulation of gene expression. Taken together with (1) stratification through GATA1 binding and (2) the knowledge that GATA1 preferentially binds to canonical GATA motifs, this leads to a putative model for erythroid gene expression whereby GATA2 binding at canonical GATA motifs favors recruitment of GATA1 and upregulation of expression, whereas binding of GATA2 at repeats/palindromes biases against GATA1 recruitment and toward constant or downregulated expression.

The topology of a TF network highlights key candidate players and predicts circuit connections but does not reveal how these circuits behave or what their outputs are. Dynamic modeling has given insights into circuit behavior and its potential impact on cell states in hematopoietic cells (Chickarmane et al., 2009; Huang et al., 2007; Narula et al., 2010; Roeder and Glauche, 2006) but has largely been restricted to well-characterized circuits; the GATA1-PU.1 paradigm provides an example. Here we used dynamic modeling to include GATA2 in this paradigm. The novel approach used does not simply model the output of a known architecture but infers the logic of regulatory interactions between TFs, by incorporating topology and dynamic binding behavior derived from TF-binding data and using high-resolution gene expression profiles to supervise the search for the best solution. The modeling implicates GATA2 as a nodal regulator of lineage specification through its repression of PU.1, validated through functional experiments in multipotent cells. Integrating new dynamic binding data for additional TFs should allow expansion of the GATA1-GATA2-PU.1 kernel to generate more extensive regulatory modules. More generally, the novel approach described here could be used for any cross-regulatory group of TFs for which sufficient dynamic binding and expression data are available, in order to predict regulatory logic and move stepwise toward the construction of larger transcriptional networks.

## Experimental Procedures

### FDCPmix Culture

FDCPmix cells were maintained in Fischer’s medium with 2% IL-3-conditioned medium and 20% horse serum. For differentiation, cells were cultured in Iscove’s modified Dulbecco’s medium plus 10% FCS and low IL-3 supplemented with either Epo and hemin (erythroid output) or G-CSF and SCF (neutrophil output). See the Supplemental Experimental Procedures for additional details.

### Primary Cell Harvest and Isolation

Primary murine bone marrow cells were harvested and FACsorted as previously reported (Pina et al., 2012).

### Chromatin Immunoprecipitation

FDCPmix cells were cross-linked with 1% formaldehyde and sonicated to yield chromatin of 100–500 bp. ChIP was performed by standard procedures using antibodies from Santa Cruz against GATA1 (sc1234x and sc265x), GATA2 (sc9008x), PU.1 (sc352x), and nonspecific rabbit IgG (Millipore; 12-370). Analysis of MPCs utilized FACS-purified kit^+^Gr-1^−^ cells. Twenty nanograms of DNA was amplified and single end sequenced at 36 bp, and reads were mapped to the mouse genome (mm9) using Bowtie (Langmead et al., 2009). Peaks were detected against rabbit IgG control using MACS (Zhang et al., 2008) and PeakRanger (Feng et al., 2011). Peaks in different experiments were called as the same bound region if the summits fell within 70 bp. To identify peaks bound in one experiment but not another, we defined “nonbound” as the absence of a MACS call in the nonfiltered list within 1 kb of that location. Motif discovery used CisFinder (Sharov and Ko, 2009) and MEME (Machanick and Bailey, 2011) with default parameters; specific motifs were mapped back to peaks using Fuzznuc (Rice et al., 2000). Peaks were assigned to the nearest transcription start site using CisGenome (Ji et al., 2008). Binary wig files were made and viewed in GBrowse (http://gmod.org) and UCSC (Kent et al., 2002).

### Lentiviral Constructs and Packaging

*GATA1ERT* and *Pu.1ERT* were subcloned into the pHR-SIN-CSGWEmGFP lentiviral expression construct under control of the SFFV promoter. *Gata2* and *Pu.1* shRNAs were subcloned into Lentilox 3.7. Recombinant plasmids were packaged essentially by published procedures. See the Supplemental Experimental Procedures for additional details.

### GATA1ERT and PU.1ERT Experiments

For gene expression analysis, triplicate samples of FDCPmix cells in self-renewal conditions were transduced with lentiviruses encoding GATA1ERT or PU.1ERT fusion proteins linked to *ires-GFP*, with empty virus as a control. GFP^+^ cells were sorted after 3 days and expanded for a further 7 days, before addition of 2 μM 4OH-tamoxifen. Cells were harvested after 0 and 24 hr of induction, and total RNA was analyzed by microarray. ChIP-seq of GATA1ERT cells utilized a subclone of FDCPmix cells stably expressing the GATA1ERT fusion protein and cultured and induced as described (Heyworth et al., 1999).

### GATA2 and PU.1 Knockdown

Triplicate samples of FDCPmix cells in self-renewal conditions were transduced with lentiviruses encoding shRNA against *Gata2* or *Pu.1*, with empty virus as a control. Five days later, GFP^+^ cells were isolated by FACsorting, lysed in TRIzol, and analyzed by microarray.

### RNA Isolation and Microarray Analysis

Total RNA was isolated using TRIzol, and microarray analysis was performed with Whole Mouse Gene Expression Microarrays (Agilent; see Supplemental Experimental Procedures). Arrays were normalized and differentials were identified with LIMMA (Smyth, 2004) and SAM (Tusher et al., 2001). Clustering of the time course was performed using k-means (http://www.r-project.org). Correspondence analysis and enrichment analysis were implemented in R (http://www.r-project.org), and hive plots were made using the HiveR package (http://academic.depauw.edu/∼hanson/HiveR/HiveR.html).

## Accession Numbers

All data have been deposited in the Gene Expression Omnibus under GEO series accession number GSE49991. The MySQL database can be accessed at https://hedberg.molbiol.ox.ac.uk/ChIPExB.

## Supplemental Information

Document S1. Supplemental Experimental Procedures, Figures S1–S6, and Table S6

Table S1. Enrichments of ChIP-Seq Target Genes within Erythroid and Neutrophil Gene Expression Clusters, Related to Figure 5 False discovery rates (FDRs) are given for enrichments of target genes identified in each ChIP-seq experiment within gene expression clusters derived by k-means clustering of erythroid and neutrophil differentiation time courses. FDRs of <0.05 are regarded as significant and are highlighted.

Table S2. GATA2 MP Peaks Subdivided by Additional Binding Behaviors Are Associated with Particular Gene Expression Clusters, Related to Figure 5 Peaks bound by GATA2 in MP cells were subdivided according to their binding in a second ChIP-seq experiment before enrichment analysis of the linked genes versus gene expression clusters derived by k-means clustering of erythroid and neutrophil differentiation time courses. FDRs of enrichments are given; FDRs of <0.05 are regarded as significant and are highlighted.

Table S3. GATA2 MP Peaks Subdivided by Motif Content Are Associated with Erythroid Gene Expression Clusters, Related to Figure 5 Peaks bound by GATA2 in MP cells were subdivided according to their DNA motif content before enrichment analysis of the linked genes versus gene expression clusters derived by k-means clustering of the erythroid differentiation time course. FDRs of enrichments are given; FDRs of <0.05 are regarded as significant and are highlighted.

Table S4. GATA2 MP Peaks Subdivided by Motif Content Are Associated with Neutrophil Gene Expression Clusters, Related to Figure 5 Peaks bound by GATA2 in MP cells were subdivided according to their DNA motif content before enrichment analysis of the linked genes versus gene expression clusters derived by k-means clustering of the neutrophil differentiation time course. FDRs of enrichments are given; FDRs of <0.05 are regarded as significant and are highlighted.

Table S5. Enrichments of ChIP-Seq Target Genes within Primary Cell Gene Expression Clusters, Related to Figure 5 False discovery rates are given for enrichments of target genes identified in each ChIP-seq experiment within gene expression clusters derived by k-means clustering of primary cell expression data. FDRs of <0.05 are regarded as significant and are highlighted in red, and significantly depleted clusters are in green.
