# Supplementary material for: Dynamic Analysis of Gene Expression and Genome-wide Transcription Factor Binding during Lineage Specification of Multipotent Progenitors
Source: Cell Stem Cell. 2013 Dec 5;13(6):754–68. doi: 10.1016/j.stem.2013.09.003 (PMC3878573; doi:10.1016/j.stem.2013.09.003)
Supplement: Document S1. Supplemental Experimental Procedures, Figures S1–S6, and Table S6 [file mmc1.pdf]

Cell Stem Cell, volume *13*

Supplemental Information

Dynamic Analysis of Gene Expression and Genome-wide Transcription Factor Binding during Lineage Specification of Multipotent Progenitors

Gillian May, Shamit Soneji, Alex J. Tipping, Jose Teles, Simon J. McGowan, Mengchu Wu, Yanping Guo, Cristina Fugazza, John Brown, Goran Karlsson, Cristina Pina, Victor Olariu, Stephen Taylor, Daniel G. Tenen, Carsten Peterson, and Tariq Enver

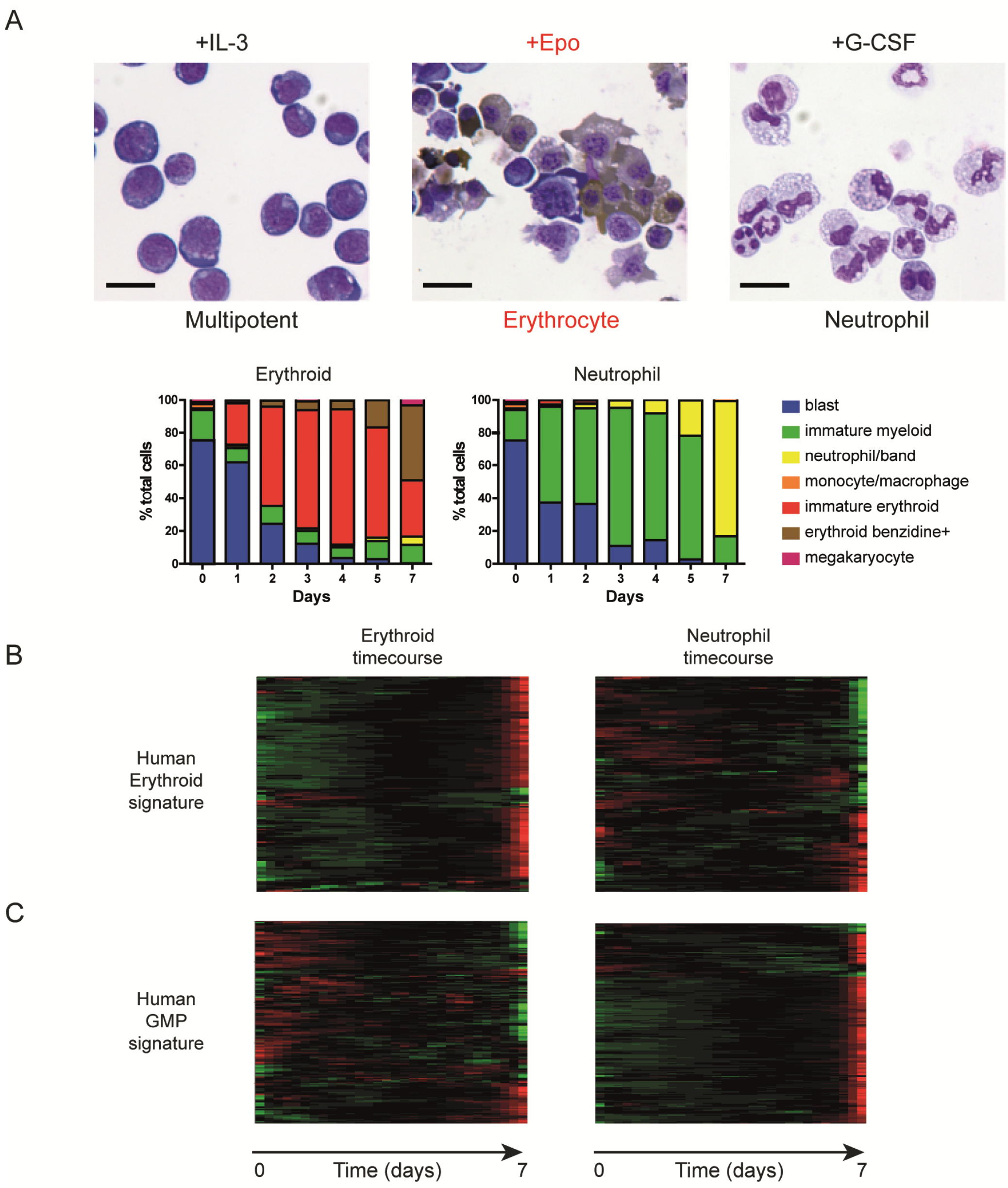

D

Factor & cell	FDCPmix data	Shared peaks	Published peaks	Same Ab?	Published cell type	Publication reference
Gata1 E	2553 (46% shared)	**1167**	12747 (9% shared)	No	Megakaryocytes (Gata1-G1ME)	Dore *et al* 2012
Gata1 E	2553 (46% shared)	**1169**	8588 (14% shared)	Yes*	C88 MEL	Soler *et al* 2010
Gata1 E	2553 (39% shared)	**996**	14218 (7% shared)	No	G1E-Gata1ER	Cheng *et al* 2009
Gata2 MP	3836 (40% shared)	**1546**	9234 (17% shared)	Yes	HPC7 cells	Wilson *et al* 2010
Gata2 E	1641 (35% shared)	**583**	18149 (3% shared)	Yes	Megakaryocytes (Gata1-G1ME)	Dore *et al* 2012
Pu.1 MP	29031 (33% shared)	**9533**	22743 (42% shared)	Yes	HPC7 cells	Wilson *et al* 2010
Pu.1 E	10088 (44% shared)	**4474**	17688 (25% shared)	Yes	ES-EP cells	Wontakal *et al* 2011
Pu.1 N	34047 (57% shared)	**19485**	45619 (43% shared)	Yes	Macrophage	Heinz *et al* 2010

Figure S1

Figure S1: Cell morphologies, expression of signature genes during differentiation timecourse, and overlap between FDCPmix ChIPSeq and published datasets (relates to Figure 1).

A. Representative images of multipotent, erythroid and neutrophil cultures at day 0 and day 7 from the timecourse (upper), stained with May-Grunwald/Giemsa and o-dianisidine. Cell morphologies were checked and scored daily; the percentages of different cell types are shown (lower).
B. Expression through the erythroid (left) and neutrophil (right) timecourses of genes defined as constituting an erythroid expression signature in human primary hematopoietic cells. (Novershtem et al., 2011).
C. Expression through the erythroid (left) and neutrophil (right) timecourses of genes defined as constituting a granulocyte-monocyte progenitor expression signature in human primary hematopoietic cells (Novershtem et al., 2011).
D. Table comparing FDCPmix ChIPSeq datasets against published datasets for GATA1, GATA2 and PU.1 generated in similar cell compartments.

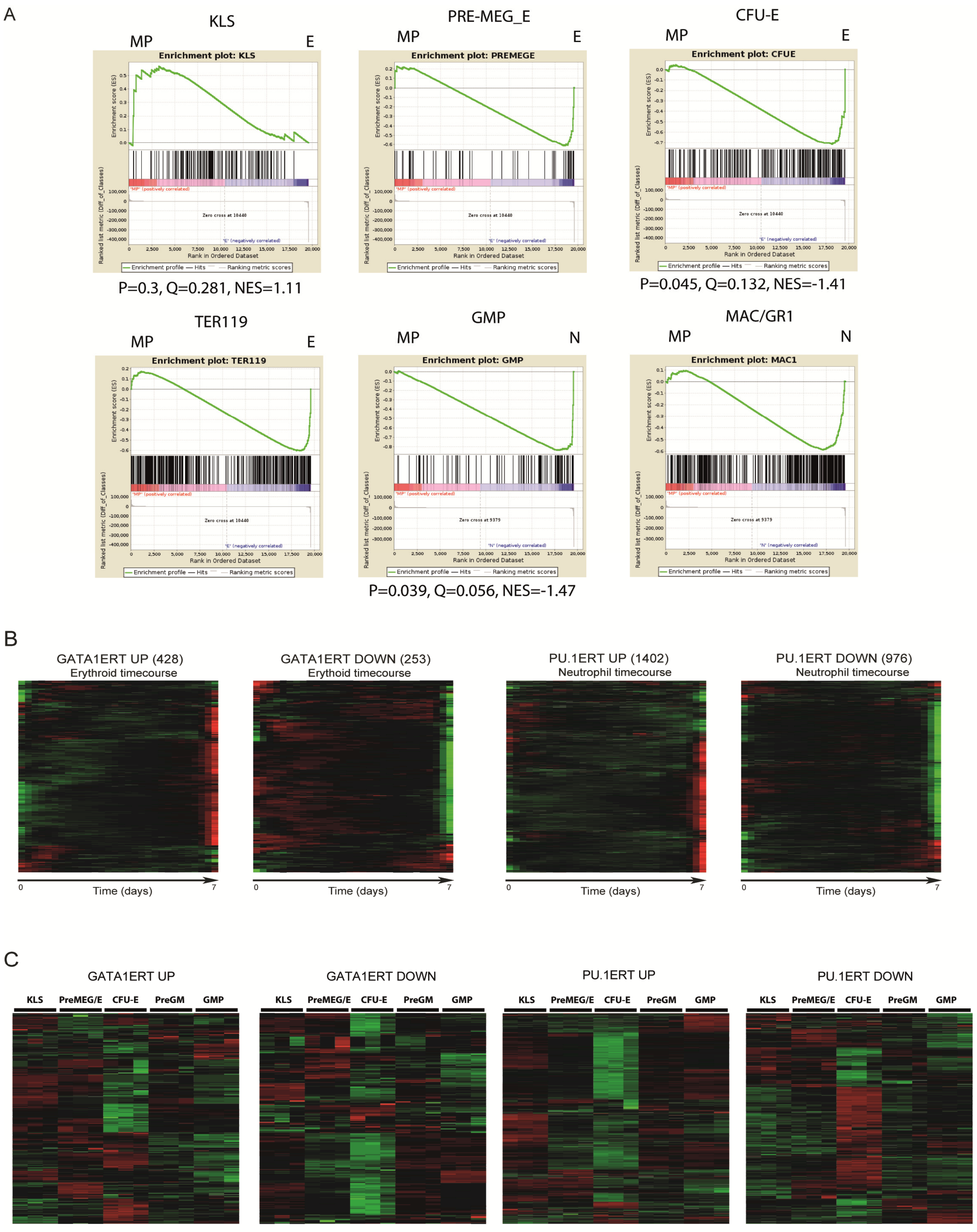
A
KLS
MP
E
Enrichment plot: KLS
P=0.3, Q=0.281, NES=1.11
PRE-MEG_E
MP
E
Enrichment plot: PREMEGE
CFU-E
MP
E
Enrichment plot: CFUE
P=0.045, Q=0.132, NES=-1.41
TER119
MP
E
Enrichment plot: TER119
GMP
MP
N
Enrichment plot: GMP
P=0.039, Q=0.056, NES=-1.47
MAC/GR1
MP
N
Enrichment plot: MAC1
B
GATA1ERT UP (428)
Erythroid timecourse
GATA1ERT DOWN (253)
Erythoid timecourse
PU.1ERT UP (1402)
Neutrophil timecourse
PU.1ERT DOWN (976)
Neutrophil timecourse
Time (days)
C
GATA1ERT UP
GATA1ERT DOWN
PU.1ERT UP
PU.1ERT DOWN
KLS
PreMEG/E
CFU-E
PreGM
GMP

Figure S2

Figure S2: Cytokine-driven differentiation versus primary murine hematopoietic cells and TF-driven differentiation of FDCPmix (relates to Figure 1).

A. Gene Set Enrichment Analysis plots showing signature genes for KLS, PreMegE, CFUe, Ter119$^+$, GMP and Mac1$^+$Gr-1$^+$ cells against multipotent (MP), erythroid day 5 (E) and neutrophil day 5 (N) FDCPmix. *p, q,* and normalized enrichment scores (NES) are shown for the subfraction most closely matching the FDCPmix MP, E and N samples.
B. Behavior of GATA1ERT-responsive genes (2-fold UP or DOWN) in the cytokine-driven erythroid differentiation of FDCPmix cells (left panels). PU.1ERT-modulated genes were compared against the neutrophil timecourse (right panels). The number of gene probes is indicated; note probes that were non-differential in the timecourse are also included here.
C. Expression in primary murine bone marrow compartments of GATA1ERT and PU.1ERT modulated genes (defined as in Figure S2B)

A

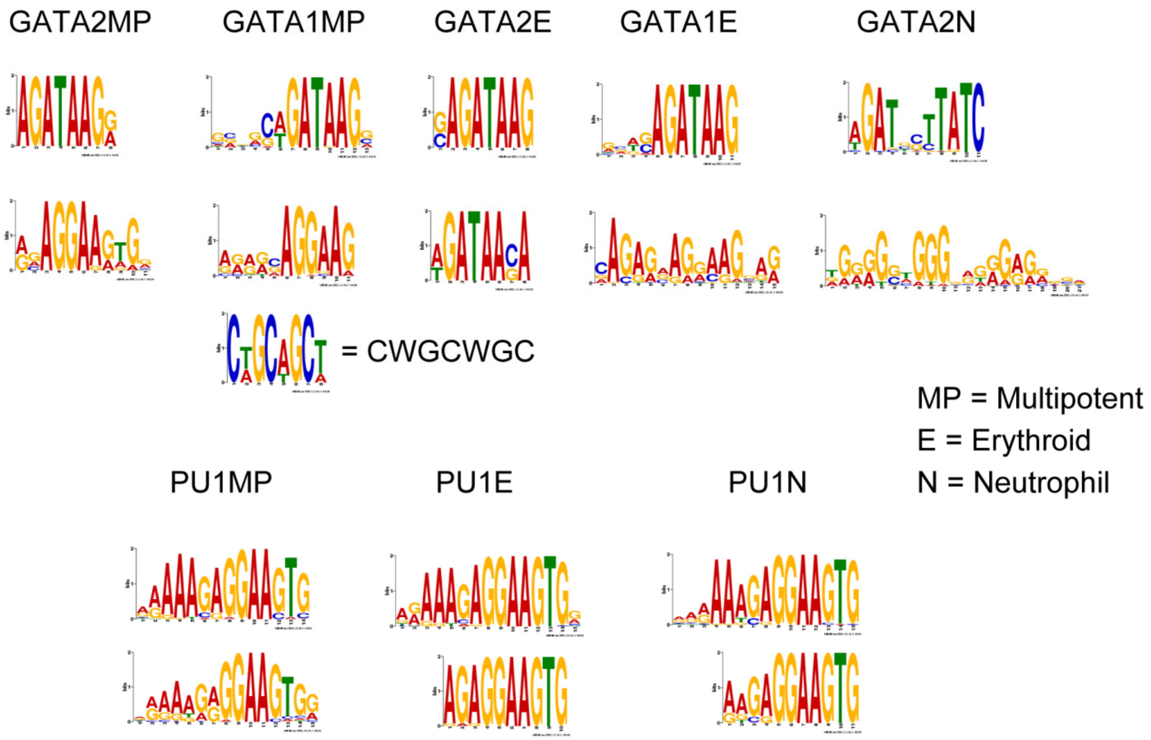
GATA2MP
GATA1MP
GATA2E
GATA1E
GATA2N
= CWGCWGC
MP = Multipotent
E = Erythroid
N = Neutrophil
PU1MP
PU1E
PU1N

B

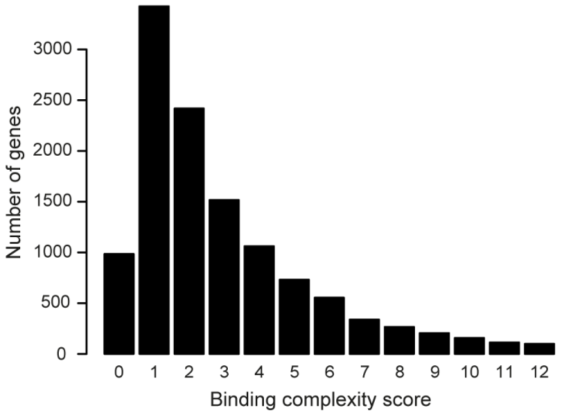
Number of genes
3000
2500
2000
1500
1000
500
0
0 1 2 3 4 5 6 7 8 9 10 11 12
Binding complexity score

Figure S3

Figure S3: Motif discovery using MEME (relates to Figure 2)

A. High scoring motifs identified in each ChIPSeq dataset by MEME are shown. MP, multipotential cells; E, erythroid cells; N, neutrophil cells. Note the identification of an E box motif (CWGCWGC) in peaks bound by GATA1 in MP cells that was not detected by CisFinder.
B. Global analysis of binding complexity. Dynamic binding at each bound location was assessed by adding a score of 1 for each gain/loss of TF binding in transiting from MP to E or N cells. Summing this for all peaks linked to a gene gives a "complexity score" for that gene. X axis; binding complexity score. Y axis; number of genes. 95% of genes are captured in the 0-12 range of complexity shown. *Gfi1b* scores 4 on this measure.

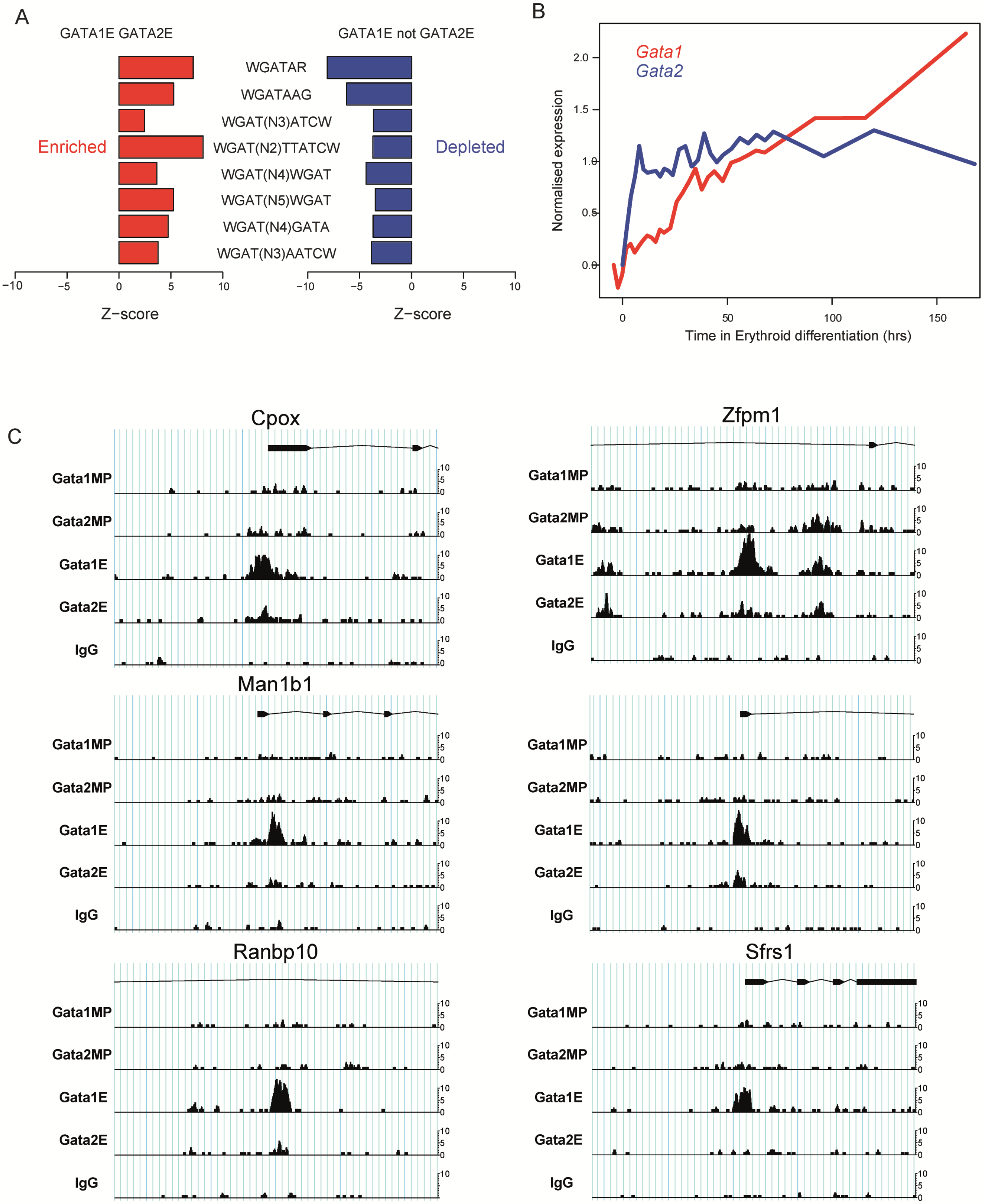
A
GATA1E GATA2E
GATA1E not GATA2E
WGATAR
WGATAAG
WGAT(N3)ATCW
WGAT(N2)TTATCW
WGAT(N4)WGAT
WGAT(N5)WGAT
WGAT(N4)GATA
WGAT(N3)AATCW
Enriched
Depleted
Z−score
Z−score
B
Gata1
Gata2
Normalised expression
Time in Erythroid differentiation (hrs)
C
Cpox
Zfpm1
Man1b1
Ranbp10
Sfrs1
Gata1MP
Gata2MP
Gata1E
Gata2E
IgG

Figure S4

Figure S4: GATA factor dynamism during erythroid differentiation (relates to Figure 3).

A. Peaks that are either co-bound by GATA1 and GATA2 in erythroid cells (left) or bound by GATA1 alone (right) are relatively enriched and depleted, respectively, in all GATA motifs analysed. Z-scores relative to the motif content of all GATA1E bound peaks.
B. Expression profiles of *Gata1* (red) and *Gata2* (blue) during 7 days (168 h) of erythroid differentiation, normalized to the expression observed in multipotent cells (0 hrs).
C. Further examples of *de novo* GATA1 binding in erythroid cells, showing absence of prior binding of GATA1 and GATA2 in MP cells and little or no binding of GATA2 in E cells.

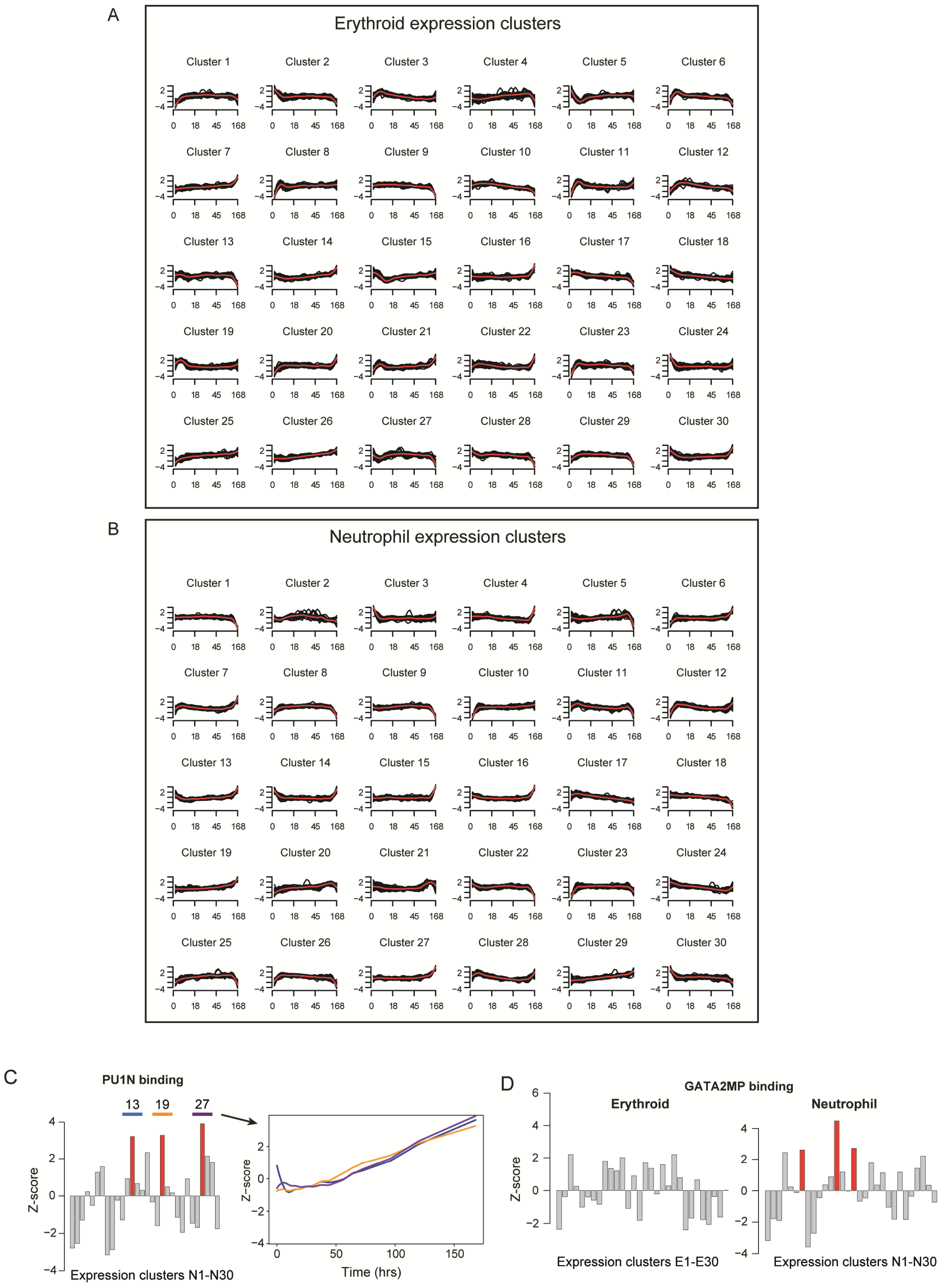
A
Erythroid expression clusters
Cluster 1
Cluster 2
Cluster 3
Cluster 4
Cluster 5
Cluster 6
Cluster 7
Cluster 8
Cluster 9
Cluster 10
Cluster 11
Cluster 12
Cluster 13
Cluster 14
Cluster 15
Cluster 16
Cluster 17
Cluster 18
Cluster 19
Cluster 20
Cluster 21
Cluster 22
Cluster 23
Cluster 24
Cluster 25
Cluster 26
Cluster 27
Cluster 28
Cluster 29
Cluster 30
2
-4
0 18 45 168
B
Neutrophil expression clusters
Cluster 1
Cluster 2
Cluster 3
Cluster 4
Cluster 5
Cluster 6
Cluster 7
Cluster 8
Cluster 9
Cluster 10
Cluster 11
Cluster 12
Cluster 13
Cluster 14
Cluster 15
Cluster 16
Cluster 17
Cluster 18
Cluster 19
Cluster 20
Cluster 21
Cluster 22
Cluster 23
Cluster 24
Cluster 25
Cluster 26
Cluster 27
Cluster 28
Cluster 29
Cluster 30
2
-4
0 18 45 168
C
PU1N binding
13
19
27
4
2
0
-2
-4
Z-score
Expression clusters N1-N30
2
0
-2
-4
Z-score
0 50 100 150
Time (hrs)
D
GATA2MP binding
Erythroid
6
4
2
0
-2
Z-score
Expression clusters E1-E30
Neutrophil
4
2
0
-2
-4
Expression clusters N1-N30

Figure S5

Figure S5: Linking gene expression to DNA binding (relates to Figure 5)

- **A.** Genes co-expressed over 7 days of erythroid differentiation were identified by k-means clustering. These clusters demonstrate the many diverse and complex patterns of gene expression that co-exist. The number of clusters was pre-defined as 30.
- **B.** Clustering of genes co-expressed over 7 days of neutrophil differentiation; clustering performed as in A.
- **C.** Enrichment analysis of PU.1 binding in neutrophils versus neutrophil expression clusters (left). Significant enrichments (z-scores) are colored red (FDR<0.05), all FDRs are given in Table S1. Centroids of significantly associated expression clusters (clusters 13, 19 and 27) are shown (right). Expression profiles for all clusters can be seen in Panels A and B.
- **D.** Enrichment analysis of GATA2 binding in MP cells versus erythroid and neutrophil expression clusters, plotted as in C. GATA2 binding in multipotent cells is not enriched in any erythroid gene expression cluster. Significant enrichments (z-scores) are colored red (FDR<0.05), all FDRs are given in Table S1. Expression profiles for all clusters can be seen in Panels A and B.

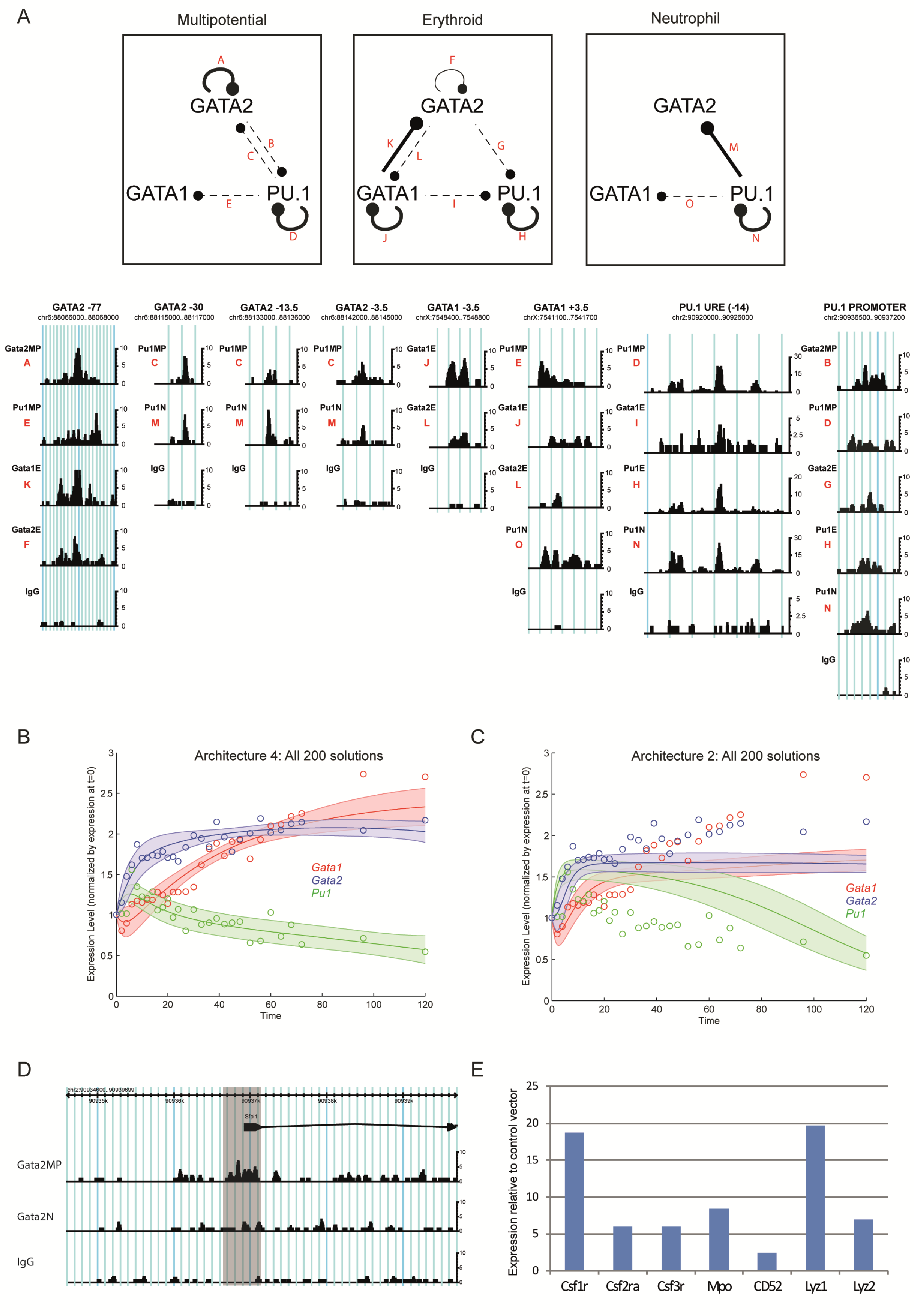
A
Multipotential
Erythroid
Neutrophil
GATA2
GATA1
PU.1
GATA2 -77
GATA2 -30
GATA2 -13.5
GATA2 -3.5
GATA1 -3.5
GATA1 +3.5
PU.1 URE (-14)
PU.1 PROMOTER
Gata2MP
Pu1MP
Gata1E
Gata2E
Pu1N
Pu1E
IgG
B
Architecture 4: All 200 solutions
C
Architecture 2: All 200 solutions
Expression Level (normalized by expression at t=0)
Time
Gata1
Gata2
Pu1
D
Sfpi1
Gata2MP
Gata2N
IgG
E
Expression relative to control vector
Csf1r
Csf2ra
Csf3r
Mpo
CD52
Lyz1
Lyz2

Figure S6

Figure S6: Experimental basis and testing of network architectures (relates to Figure 6).

A. Binding summary for GATA1, GATA2 and PU.1 over their own and each other's loci, based on the ChIPSeq data. Bold connectors, strong enrichments; solid connectors, intermediate enrichments; dotted connectors, weak enrichments. ChIPSeq binding data from the *Gata2*, *Gata1* and *Pu.1* loci that supports interactions A to O is also shown. Note that a single binding interaction in the summary figure (A to O) may reflect binding at more than one location within a locus.

B. Example of a good fit to the time-course gene expression profiles, using all 200 best solutions for architecture 4. Full lines, mean expression; Shaded contours, standard deviation; Circles, experimental data points; Red, *Gata1*; Blue, *Gata2*; Green, *Pu.1*.

C. Example of a poor fit to the time-course gene expression profiles, using all 200 best solutions for architecture 2. Full lines, mean expression; Shaded contours, standard deviation; Circles, experimental data points; Red, *Gata1*; Blue, *Gata2*; Green, *Pu1*. Note that the networks shown here and in Fig S6B differ solely in the nature of the GATA2/PU.1 interaction (repression in architecture 4; activation in architecture 2. See Table S6).

D. GATA2 binding to the *Pu.1* (*Sfpi1*) promoter can be seen in FDCPmix MP cells, suggesting the repression of *Pu.1* by GATA2 is direct. Note the much reduced, or absent, binding of GATA2 in N (GATA2N).

E. Upregulation of a panel of myeloid-affiliated genes in multipotent FDCPmix after lentiviral-mediated knockdown of *Gata2* expression. Expression was assessed by microarray and is shown relative to control vector.

Supplemental Tables

Architecture #	a_2 (G_1/G_2)	a_3 (G_2/G_1)	a_4 (G_2/G_2)	a_5 (G_2/P_1)	a_6 (P_1/G_2)
1	-	+	+	+	+
2	-	+	+	+	-
3	-	+	+	-	+
4	-	+	+	-	-
5	-	+	-	+	+
6	-	+	-	+	-
7	-	+	-	-	+
8	-	+	-	-	-
9	-	-	+	+	+
10	-	-	+	+	-
11	-	-	+	-	+
12	-	-	+	-	-
13	-	-	-	+	+
14	-	-	-	+	-
15	-	-	-	-	+
16	-	-	-	-	-
17	+	+	+	+	+
18	+	+	+	+	-
19	+	+	+	-	+
20	+	+	+	-	-
21	+	+	-	+	+
22	+	+	-	+	-
23	+	+	-	-	+
24	+	+	-	-	-
25	+	-	+	+	+
26	+	-	+	+	-
27	+	-	+	-	+
28	+	-	+	-	-
29	+	-	-	+	+
30	+	-	-	+	-
31	+	-	-	-	+
32	+	-	-	-	-

Table S6

Supplementary Table Legends

Table S1 Enrichments of ChIPSeq target genes within erythroid and neutrophil gene expression clusters (relates to Figure 5).

False discovery rates are given for enrichments of target genes identified in each ChIPSeq experiment within gene expression clusters derived by k-means clustering of the erythroid and neutrophil differentiation timecourses. FDRs of < 0.05 are regarded as significant and are highlighted.

Table S2 GATA2 MP peaks subdivided by additional binding behaviors are associated with particular gene expression clusters (relates to Figure 5).

Peaks bound by GATA2 in MP cells were subdivided according to their binding in a second ChIPSeq experiment before enrichment analysis of the linked genes versus gene expression clusters (derived by k-means clustering of the erythroid and neutrophil differentiation timecourses). FDRs of enrichments are given; FDRs of < 0.05 are regarded as significant and are highlighted.

Table S3 GATA2 MP peaks subdivided by motif content are associated with erythroid gene expression clusters (relates to Figure 5).

Peaks bound by GATA2 in MP cells were subdivided according to their DNA motif content before enrichment analysis of the linked genes versus erythroid gene expression clusters (derived by k-means clustering of the erythroid differentiation timecourse). FDRs of enrichments are given; FDRs of < 0.05 are regarded as significant and are highlighted.

Table S4 GATA2 MP peaks subdivided by motif content are associated with neutrophil gene expression clusters (relates to Figure 5).

Peaks bound by GATA2 in MP cells were subdivided according to their DNA motif content before enrichment analysis of the linked genes versus neutrophil gene expression clusters (derived by k-means clustering of the neutrophil differentiation timecourse). FDRs of enrichments are given; FDRs of < 0.05 are regarded as significant and are highlighted.

Table S5 Enrichments of ChIPSeq target genes within primary cell gene expression clusters (relates to Figure 5).

False discovery rates are given for enrichments of target genes identified in each ChIPSeq experiment within gene expression clusters derived by k-means clustering of the primary cell gene expression data. FDRs of < 0.05 are regarded as significant and are highlighted in red, and significantly depleted clusters in green.

Table S6 Possible Architectures within the GATA2/GATA1/PU.1 Sub-network (relates to Figure 6).

All possible combinations of molecular interactions within the GATA2/GATA1/PU.1 sub-network, where "+" and "-" represent positive and negative regulation respectively. Directionality of interactions is denoted by the order of the genes in each pair (e.g. G1/G2 denotes GATA1 binding to GATA2). Best set of estimated parameter values for architecture 4: a_{01}=0.076, a_{11}=0.044, a_{02}=0.059, a_{12}=0.019, a_{03}=0.059, a_{13}=0.048, a_{04}=17.461, a_{14}=0.001, a_5=24.205, a_{06}=12.175, a_{16}=0.004, a_7=27.953, a_{08}=0.074, a_{18}=0.039, a_{09}=17.521, a_{19}=0.036, b=26.358, γ=0.375, E=0.500

Supplemental Experimental Procedures

FDCPmix cell culture

FDCPmix cells were maintained in modified Fischer's medium (Gibco 041-95170 M) supplemented with 20% batch-tested horse serum (PAA), 2% IL3-conditioned medium, 2mM L-glutamine and 1% penicillin/streptomycin at a density of $5x10^4$ to $8x10^5$ cells/ml. For the gene expression timecourse, cells were washed and seeded at 10^5 cells/ml, with 3 replicate flasks per timepoint. Erythroid conditions; IMDM, 10% FCS, 0.03 ng/ml IL-3 (R&D 403-ML), 10U/ml human epo (EPREX), 0.2 mM hemin (Sigma H9039), incubated at 37°C in 5% O_2/5% CO_2. Neutrophil conditions; IMDM, 10% FCS, 0.03 ng/ml mouse IL-3 (R&D 403-ML), 10^5 U/ml human G-CSF (Neupogen), 100 ng/ml mouse SCF (R&D 455-MC) incubated at 37°C in 5% CO_2.

Chromatin Immunoprecipitation Assay

10^8 FDCPmix cells were incubated in DMEM plus 1% formaldehyde for 15 minutes at room temperature with gentle stirring. The reaction was quenched by addition of 0.125M glycine, and the incubation continued for 5 minutes. Cells were harvested at 4°C and washed in cold PBS. Cells were harvested and the pellet resuspended in 1.5x pellet volume in cold cell lysis buffer (CLB: 10 mM Tris-HCl pH 8.0, 10 mM NaCl, 0.2% NP40, 10mM sodium butyrate, 50 µg/ml PMSF, 1 µg/ml leupeptin) and incubated on ice for 10 minutes. Nuclei were harvested at 600g for 5 minutes at 4°C and resuspended in 0.5 ml cold nuclear lysis buffer (NLB: 50 mM Tris-HCl pH 8.0, 10 mM EDTA, 1% SDS, 10mM sodium butyrate, 50 µg/ml PMSF, 1 µg/ml leupeptin) and incubated on ice for 10 minutes before addition of 0.3 ml of IP dilution buffer (IPDB: 20mM Tris-HCl pH 8.0, 150 mM NaCl, 2mM EDTA, 1% Triton X-100, 0.01% SDS, 10mM sodium butyrate, 50 µg/ml PMSF, 1 µg/ml leupeptin). Cells were transferred to a 15 ml polystyrene tube and sonicated on a high-setting (Bioruptor, Diagenode) on ice-water for 10 minutes (30s on, 30s off). The sheared chromatin was transferred to a micro-centrifuge tube and spun at 16,000 g for 5 minutes at 4ºC. The volume was measured as the chromatin was transferred to a clean 15 ml polypropylene tube and the final volume adjusted to 6 ml using a 5:3 mixture of NLB:IPDB. Chromatin was usually snap-frozen at this stage in liquid nitrogen and stored at -80°C. On thawing, the chromatin was pre-cleared with 200 µl Protein G-agarose beads (Roche) for 3h at 4°C with rotation. The beads were removed by centrifugation at 1800 g for 2 minutes at 4°C. The chromatin was transferred to a clean 15 ml polypropylene tube and an equal volume of modified IPDB added (a 1:4 mixture of NLB and IPDB). An input sample was removed and the remainder of the chromatin divided equally between eight 2 ml tubes. 10 µg of antibody was added to each tube and incubated overnight at 4°C with rotation. Chromatin/Ab was centrifuged at 16000 g for 5 minutes at 4°C and transferred to a clean tube. 100 µl of Protein G agarose beads, pre-blocked by incubation with 10 mg/ml BSA, were added to the chromatin/ antibody solution and incubation continued for 3h at 4°C with rotation. The beads were harvested at 5000 g for 2 minutes at 4°C and washed with 750 µl low salt buffer (20 mM Tris-HCl pH 8.0, 50 mM NaCl, 2 mM EDTA, 1% Triton X-100, 0.1% SDS). The beads were transferred to a clean tube and the wash repeated, followed by washing once with LiCl buffer (10 mM Tris-HCL pH 8.0, 250 mM LiCl, 1 mM EDTA, 1% NP-40, 1% deoxycholic acid) and twice with TE, pH 8.0. All washes used 750 µl buffer at 4°C. The complexes were eluted from the beads by adding 200 µl elution buffer (100 mM $NaHC0_3$, 1% SDS) and mixing for 10 minutes at room temperature on a Thermomixer at 650 rpm before spinning at 5000 g for 2 minutes. The elution was repeated and the two eluates pooled. Cross-links were reversed by adding 0.3M NaCl and 5 µg/ml RNase A and incubating overnight at 65°C. Samples were treated with 200 µg/ml Proteinase K at 45°C for at least 4 hours. DNA was purified by phenol/chloroform extraction and ethanol precipitation using 20 µg glycogen as carrier.

To improve recovery of GATA1, a mixture of 2 antibodies (10 μg of each of sc1234x and sc265x) was used. Recovery of rat monoclonal sc265x was enhanced by use of 100 μl rabbit anti-rat IgG (Jackon ImmunoResearch, 312-005-003) pre-bound to Protein A agarose beads (Johnson et al, 2002) in addition to 100 μl Protein G agarose beads.

ChIPSeq

ChIP DNA was quantified using Quant-iT PicoGreen dsDNA Assay Kit (cat# P7589 Invitrogen, Carlsbad, CA, USA). 10-20 ng of ChIP DNA was used to construct sequencing libraries using either ChIP-Seq sample preparation kit (cat# IP-102-1001, Illumina, San Diego, CA, USA) as per manufacture's instruction or individual reagents supplied by New England Biolabs. Constructed libraries were subjected to a final size-selection step on 10% Novex TBE Gels (cat# EC6275BOX, Invitrogen, Carlsbad, CA, USA) or 2% TAE agarose gels. DNA fragments of 200-400bp were excised from SYBR-green-stained gels. DNA was recovered from the gel and assessed using the BioAnalyzer 2100 (Agilent) and Illumina's qPCR qantification protocol. Quantified ChIP DNA libraries were then sequenced using the single-end sequencing protocol at either 36bp or 50bp. Image analysis and base calls were performed by Illumina software suite with default settings.

RNA isolation and microarray analysis

Total RNA was isolated using TRIzol reagent, and RNA concentration and integrity determined using RNA 6000 Nano RNA kit (Agilent) on BioAnalyzer 2100 (Agilent). Total RNA (200-250 ng) and Agilent One-Color RNA Spike-In were reverse-transcribed and linearly amplified in the presence of Cy3-labeled CTP using Low RNA Input One-Color Kit (Agilent) following Agilent protocols, to generate Cy3-labeled cRNA for hybridisation to high-density microarrays. Quality and yield/specific activity of cRNA were determined using RNA 6000 Nano kit and spectrophotometer (NanoDrop ND-1000). All samples generated comparable quality cRNA profiles.1.6μg Cy3-labeled cRNA was hybridized to an individual 44K sub-array of 4x44K high-density microarray slide (Whole Mouse Gene Expression Microarrays, Agilent), washed, scanned and feature-extracted as per Agilent protocols.

Lentiviral constructs

FLAG-tagged GATA1ERT (Heyworth et al., 2002) was excised from pBluescript with BamHI and SalI and subcloned into the cloning vector pSIN-BX-IR/EMW. The entire FLAG-GATA1ERT-ires-EmGFP insert was then excised with BamHI and NotI and ligated to pHR-SIN-CSGW cut with BamHI and NotI. Expression is under the control of the SFFV promoter.

PU1ERT was subcloned from pBABEpuro-mPU1ERT (Wang et al.,1999) using BsmBI and SphI. The fragment was treated with Klenow enzyme and inserted into the BamHI site of pHR-SIN-CSGW-ires-EmGFP (Demaison et al, 2002).

To generate GATA2 and PU1 knockdown viruses, primer sequences given below were annealed slowly pairwise to yield DNA fragments with ends complementary to the cut HpaI and XhoI sites of pLentilox 3.7. Primer sequences were (5' to 3'):

shGata2_PLUS

TGTGTCACTCTCCGAGAGCATGGGATCCGATGCTCTCGGAGAGTGACACTTTTTC

shGata2_MINUS

TCGAGAAAAAGTGTCACTCTCCGAGAGCATCGGATCCCATGCTCTCGGAGAGTGACACA

shPu1_PLUS

TGCCATAGCGATCACTACTGGGGGATCCGCCCAGTAGTGATCGCTATGGCTTTTTC

shPu1_MINUS

TCGAGAAAAAGCCATAGCGATCACTACTGGGCGGATCCCCCAGTAGTGATCGCTATGGCA

Recombinant plasmids were packaged into lentiviral particles essentially as described (Woods et al, 2001). FDCPmix cells were transduced at an MOI of between 50 and 200.

Analysis of Gene Expression data

Data was acquired using feature extraction software (Agilent Technologies) from which the intensities were measured. Surface detrended intensities were normalized using four rounds of cyclic loess (Ballman et al., 2009). Differentially expressed genes were identified using two methods LIMMA (Smyth., 2004) and SAM (Tusher et al., 2001). FDR and B-values were obtained for all pairwise comparisons of timepoints, and for each probe the maximum B-value and minimum FDR was recorded. For SAM, a multiclass analysis was performed where the triplicates of each time point were grouped as a class. For each probe the q-value was calculated. Probes were deemed differential if B>10 and/or q <10. For steady-state data, LIMMA was used to find differentially expressed genes. GEDI plots were made using GEDI (Eichler et al., 2003) using a 30x25 and 8x8 grid respectively. Clustering of the FDCPmix differentiation expression data was performed on the median of replicates using k-means into 30 partitions, post z-score normalization and profile smoothing. Genes responding to ERT fusions were defined as 2-fold up- or down-regulated if their expression changed 2-fold more over 24h in response to tamoxifen than in cells infected with control virus and treated in parallel. Primary cell signature genes were defined using LIMMA (B>10) and clustered using kmeans into 12 partitions.

To position FDCPmix multipotential, erythroid and neutrophil cells against primary hematopoietic cells in terms of their gene expression, MP, E and N signatures were derived from FDCPmix using LIMMA (B>2,>2-fold change). These were used as gene sets to compare against all pairwise comparisons of primary cell samples (KLS, PreMegE, CFUe, PreGM, GMP, Ter119^{+}, Mac1^{+}Gr-1^{+}) in a gene set enrichment analysis (GSEA). For a given comparison between two primary cell compartments, GSEA was performed and a vote was given to a compartment if enriched for an FDCPmix signature gene set (FDR<25%). After all comparisons were made, the number of votes cast to each primary cell compartment was totalled.

Peak Calling and Motif Analysis Analysis

Reads were mapped to a repeat-masked version the mouse genome (mm9) using Bowtie (Langmead et al., 2009), and duplicate reads were removed. Peaks were detected against a rabbit IgG control using two methods 1) MACS (Zhang et al., 2008) (mfold=15, bw =100), and 2) PeakRanger (Feng et al., 2011) (b=100, p=0.01, t=4). High confidence peaks were called so if detected by both algorithms (summits within 250 base pairs of each other) and the fold-change by MACS ≥ 15 and p<1e-09. We called peaks as being bound in two conditions if the genomic coordinates of the summits were within 70bp of each other. We also define peaks as being bound in one condition and not another. We use a stringent definition of non-bound in the second condition as the absence of a MACS call within 1kb in the non-filtered list of MACS calls. This avoided the possibility of low-level binding which just failed to meet stringent thresholds defined above as being called truly absent, therefore removing low-confidence peaks from both analyses. Peaks were assigned to the nearest TSS using CisGenome (Hongkai et al., 2008). Binary wig files were made and viewed in Gbrowse (http://gmod.org) and UCSC.

De novo motif detection was performed using CisFinder (Sharov et al., 2009) and MEME-ChIP (Machanick et al., 2011) using default parameters. Motifs identified were converted to IUPAC codes and mapped against the sequences within bound regions using Fuzznuc (Rice et al., 2000). To determine over/under representation of motifs for a specific subset of peaks of size N, we used a resampling method where S rounds of N are drawn, and numbers of peaks containing motif M are counted at each round. A p-value for each motif is calculated by taking the number of resampled counts which exceed the original, divided by S. P-values are then corrected to account for multiple experiment testing using Bejamini-Hochberg correction as implemented in the “fdrtool” package for R.

Combinatorial data analyses

Binding complexity was determined by firstly defining all bound regions across all 8 multipotent and day 5 ChIPs combined. For each chromosome, pairwise distances were calculated between all binding events for all 8 IPs together. The resulting matrix was hierarchically clustered, and the dendrogram cut at a height where overlapping regions (optimized visually on a browser) were called as one cluster. Within each cluster, a bound region was defined as being between the most 5' and 3' co-ordinates of the constituent set. This identified 42911 regions bound by any number of TFs within the 8 ChIPSeq experiments. These 42911 regions were then compared back to the 8 IPs, for each region scoring a 1 if bound in an IP, 0 otherwise. For all peaks *X* assigned to gene *Y* we devise a simple complexity score considering the number of transitions from the multipotential state. An example is given in the table below, demonstrating how the score is calculated for three hypothetical peaks assigned to a single gene.

**Peak**	**Gata2**				**Pu1**				**Gata1**			**Total**
	MP	E	N	Score	MP	E	N	Score	MP	E	Score	
X1	0	0	1	1	1	1	1	0	1	1	0	1
X2	1	0	0	2	1	1	0	2	1	1	0	4
X3	1	1	1	0	0	1	1	2	0	1	1	3
**Total**				**3**				**4**			**1**	**8**

A total of 8 transitions are seen from the multipotent state hence a score of 8.

Gene expression clusters enriched/depleted for ChIP targets were identified by bootstrapping as described above, where bootstrapped cluster/IP entries in the contingency table were defined by the prior probabilities determined by cluster/IP size. Clusters were deemed enriched/depleted if corrected p or $1-p < 0.05$. Correspondence and cluster analysis, as all analyses, were done using the R language. Hive plot (Krzywinski et al., 2011) was made using the HiveR package.

Data storage

A MySQL database was created to store the microarray/ChIPSeq analyses, and a custom web interface written using Perl CGI to allow flexible querying of the data. The web interface allows the user to filter genes matching pre-defined statistical criteria within a single analysis set. A key aspect of the database is that all data from each analysis set has already been mapped to a central gene table consisting of the mouse gene IDs from NCBI. These gene links allow several statistical filters across different platforms to be combined in a linear manner such that only genes matching the first and all subsequent filters are identified. The results of such filter(s) can be presented on-screen in a variety of ways from simple gene lists and graphical plots, to more specific formats such as FASTA-formatted DNA sequences and Genesis (Stern et al., 2002) input format.

Computational Modelling

Model Framework : To determine the nature of the interactions within the Gata1/Pu.1/Gata2 sub-network, we use a mechanistic model of deterministic rate equations that bridges ChIPseq binding data with gene expression time series data.

We start by formulating the basic structure of the gene regulatory sub-network using the ChIPseq data for the multipotential progenitor (MP) and erythroid-differentiated (ED) states (see Figure 6A). Existing literature and the qualitative behavior of the ChIPseq data leads to the following assumptions:

(i) GATA1 and PU.1 exhibit their well-established cross-inhibition and positive autoregulation (Graf, 2002; Chickarmane et al., 2009; Graf and Enver, 2009; Narula et al., 2010).

(ii) External factor *E* (or "X" in Figure 5A) promoting GATA1 with a constant strength is present in erythroid differentiation. An example of external signal *E* could be EPO.

(iii) From our ChIPseq data, GATA2 interacts with GATA1, PU1 and itself but the exact nature of these interactions is unknown.

(iv) The dynamic binding behavior is taken into account by comparing observed binding strengths at day 0 (MP) and day 5 (ED) for each interaction.

Mathematical Formulation

From the ChIPseq data one cannot infer the nature (signs) of interactions of GATA2 with itself, GATA1 and PU1. Hence we have explored all six possible signs for these five possible interactions (Figure 6B). We then assessed which of the 32 resulting possible architectures (Table S6) are more likely to explain the observed gene expression time series behavior for the three genes. Although a similar approach has been recently followed in the study of morphogen gradient regulation (Cotterell and Sharpe, 2010), this sort of exhaustive search in combinatorial space is, to our knowledge, novel in its use of dynamic binding and gene expression data to infer the nature of regulatory interactions. For each potential gene network architecture, we derived one set of differential equations following a thermodynamic approach (Shea and Ackers, 1985; Buchler et al., 2003; Bintu et al., 2005; Narula et al., 2010). These equations have 18 unknown parameters, defining the binding strengths and decay rates, which can be estimated from gene expression time series data.

The observed changes in binding strengths are modelled as exponential increase or decay in time. For cases where no significant changes where observed in the ChIPseq data (comparing MP versus ED), the binding strength was assumed to be constant (see black line plot in Figure 6B). One example (architecture 4 in Table S6) of the equations describing the behavior of GATA1, PU.1 and GATA2 within one of the potential architectures is given by:

$$\frac{d[G_1]}{dt} = \frac{(a_1\cdot[G_1]+a_3\cdot[G_2]+b\cdot X)}{(1+a_1\cdot[G_1]+a_3\cdot[G_2]+b\cdot E+a_9\cdot[G_1]\cdot[P_1])} - \gamma\cdot[G_1]$$

$$\frac{d[P_1]}{dt} = \frac{(a_7\cdot[P_1])}{(1+a_7\cdot[P_1]+a_5\cdot[G_2]+a_8\cdot[G_1]\cdot[P_1])} - \gamma\cdot[P_1]$$

$$\frac{d[G_2]}{dt} = \frac{(a_4\cdot[G_2])}{(1+a_4\cdot[G_2]+a_2\cdot[G_1]+a_6\cdot[P_1])} - \gamma\cdot[G_2]$$

where the GATA1, PU.1, GATA2 and concentration levels are denoted by $[G_1]$, $[P_1]$ and $[G_2]$ respectively and t denotes the time. The varying binding strength parameters are parameterized as

$$a_i = a_{i0}\cdot\left(e^{(a_{i1}\cdot t)}\right), \text{ for increasing binding strengths}$$

$$a_i = a_{i0}\cdot\left(e^{-(a_{i1}\cdot t)}\right), \text{ for decreasing binding strengths}$$

with the exception of a_5 and a_7 which are constant. The degradation rates are denoted by γ and are assumed to be the same for all three genes), b represents the strength of $\boldsymbol{X}$ signaling on the system.

The parameter sets of each of the 32 proposed dynamical models θ_a, a = 1.32 were estimated from the microarray time-series data using the simulated annealing algorithm [9] to minimize the error between model predictions and observed time series data. This is an iterative process, where the parameters of each model a are updated to minimize a quadratic error E_a over 27 time points covering the time period from day 0 to day 5:

$$E_a = \sum_{t=1}^{27}\sum_{k=G_1,P_1,G_2}\left(M_a(t,k,\theta_a) - d(t,k)\right)^2$$

Here, $M_a(t, k, \theta_a)$ denotes model generated points, $d(t, k)$ represents the experimental data points normalized by expression value at time $t = 0$, a is the architecture, t is the time and k denotes the three different genes. The simulated annealing algorithm was used to find an approximation of the global minimum of E_a. In order to

screen for most likely architectures we ran the optimization protocol 200 times for each of the 32 architectures, keeping the best set of parameters i.e. the parameters that minimize E_a, each time. As an example, the best set of parameters for architecture 4 is provided in the legend of Table S6.

<u>Supplemental References</u>

Ballman, K.V., Grill D.E., Oberg, A.L., Therneau, TM. (2009). Faster cyclic loess: normalizing RNA arrays via linear models. Bioinformatics *1,* 2778-2786.

Bintu, L., Buchler, N.E., Garcia, H.G., Gerland, U., Hwa, T., Kondev J., Phillips, R. (2005) Transcriptional regulation by the numbers: models. Curr. Opin. Genet. Dev. *15,* 116-124.

Brown, M., Li, W., Liu, X.S. (2008) Model-based analysis of ChIP-Seq (MACS). Genome Biol *9,* R137.

Buchler, N.E., Gerland, U., Hwa, T. (2003) On schemes of combinatorial transcription logic Proc. Natl. Acad. Sci. U S A *100,* 5136-5141.

Cotterell, J., Sharpe, J. (2010) An atlas of gene regulatory networks reveals multiple three-gene mechanisms for interpreting morphogen gradients. Mol. Syst. Biol. *6*, 425.

Demaison, C., Parsley, K., Brouns, G., Scherr, M., Battmer, K., Kinnon, C., Grez, M., Thrasher, A.J. (2002) High-level transduction and gene expression in hematopoietic repopulating cells using a human immunodeficiency virus type 1-based lentiviral vector containing an internal spleen focus forming virus promoter. Hum. Gene Ther. *13,* 803-813

Eichler, G.S., Huang, S., Ingber, D.E. (2003) Gene Expression Dynamics Inspector (GEDI): for integrative analysis of expression profiles, Bioinformatics *19,* 2321-2322

Feng, X., Grossman, R., Stein, L. (2011). PeakRanger: A cloud-enabled peak caller for ChIP-seq data. BMC Bioinformatics *12,* 139.

Graf, T. (2002) Differentiation plasticity of hematopoietic cells. Blood *99,* 3089-3101.

Heyworth, C., Pearson, S., May, G., Enver, T. (2002) Transcription factor-mediated lineage switching reveals plasticity in primary committed progenitor cells. EMBO J *21,* 3770-3781.

Ji, H., Jiang, H., Ma, W., Johnson, D.S, Myers R.M, and Wong, W.H. (2008) An integrated software system for analyzing ChIP-chip and ChIP-seq data. Nature Biotechnology, *26,* 1293-1300.

Johnson, K.D., Grass, J.A., Boyer, M.E., Kiekhaefer, C.M., Blobel, G.A., Weiss, M.J., Bresnick, E.H. (2002). Cooperative activities of hematopoietic regulators recruit RNA polymerase II to a tissue-specific chromatin domain. PNAS USA *99,* 11760-11765.

Kirkpatrick, S., Gelatt, Jr. C. D., Vecchi, M.P. (1983) Optimization by simulated annealing. Science *220,* 671-680.

Krzywinski, M., Birol, I., Jones, S.J., Marra, M.A. (2011). Hive Plots - Rational Approach to Visualizing Networks. Briefings in Bioinformatics *Epub.*

Langmead, B., Trapnell, C., Pop, M., Salzberg, S.L. (2009) Ultrafast and memory-efficient alignment of short DNA sequences to the human genome. Genome Biol. *10:R25.*

Liew, C.W., Rand, K.D., Simpson, R.J., Yung, W.W., Mansfield, R.E., Crossley, M., Proetorius-Ibba, M., Nerlov, C., Poulsen, F.M., Mackay, J.P. (2006) Molecular analysis of the interaction between the hematopoietic master transcription factors GATA-1 and PU.1. J. Biol. Chem *281,* 28296-28306.

Machanick, P., Bailey, T.L. (2011). MEME-ChIP: motif analysis of large DNA datasets", Bioinformatics *27,* 1696-1697.

Rice, P., Longden, I., Bleasby, A. (2000). EMBOSS: the European Molecular Biology Open Software Suite. Trends Genet *16,* 276-277.

Sharov, A.A., Ko, M.S.H. (2009). Exhaustive search for over-represented DNA sequence motifs with CisFinder. DNA Research *16,* 261–273.

Shea, M.A., Ackers, G.K. (1985). The OR control system of bacteriophage λ. A physical chemical model for gene regulation. J Mol Biol *181,* 211-230.

Smyth, G. K. (2004). Linear models and empirical Bayes methods for assessing differential expression in microarray experiments. Statistical Applications in Genetics and Molecular Biology *3:1,* Article 3.

Sturn A., Quackenbush J., Trajanoski Z. (2002). Genesis: Cluster analysis of microarray data. Bioinformatics. *18,* 207-208.

Tusher, V. G., R. Tibshirani, et al. (2001). Significance analysis of microarrays applied to the ionizing radiation response. PNAS USA *98,* 5116–5121.

Wang, X., Scott, E., Sawyers, C.L., Friedman, A.D. (1999) C/EBPalpha bypasses granulocyte colony-stimulating factor signals to rapidly induce PU.1 gene expression, stimulate granulocytic differentiation, and limit proliferation in 32D cl3 myeloblasts. Blood *94,* 560-571.

Woods, N.B., Mikkola, H., Nilsson, E., Olsson, K., Trono, D., and Karlsson, S. (2001). Lentiviral-mediated gene transfer into hematopoietic stem cells. J Intern Med *249*, 339-343.
